# Cross-direct effects in settings with two mediators

**DOI:** 10.1093/biostatistics/kxac037

**Published:** 2022-09-02

**Authors:** Erin E Gabriel, Arvid Sjölander, Dean Follmann, Michael C Sachs

**Affiliations:** Section of Biostatistics, Department of Public Health, University of Copenhagen, Øster Farimagsgade 5, 1353 Køpenhavn, Denmark; Department of Medical Epidemiology and Biostatistics, Karolinska Institutet, Nobels väg 12A, Stockholm 17177, Sweden; Biostatistics Research Branch, National Institute of Allergy and Infectious Diseases, Bethesda, 5601 Fishers Lane, Rockville, MD 20892, USA; Section of Biostatistics, Department of Public Health, University of Copenhagen, Copenhagen, Denmark

**Keywords:** Causal pathways, Multiple mediation, Symbolic bounds

## Abstract

When multiple mediators are present, there are additional effects that may be of interest beyond the well-known natural (NDE) and controlled direct effects (CDE). These effects cross the type of control on the mediators, setting one to a constant level and one to its natural level, which differs across subjects. We introduce five such estimands for the cross-CDE and -NDE when two mediators are measured. We consider both the scenario where one mediator is influenced by the other, referred to as sequential mediators, and the scenario where the mediators do not influence each other. Such estimands may be of interest in immunology, as we discuss in relation to measured immunological responses to SARS-CoV-2 vaccination. We provide identifying expressions for the estimands in observational settings where there is no residual confounding, and where intervention, outcome, and mediators are of arbitrary type. We further provide tight symbolic bounds for the estimands in randomized settings where there may be residual confounding of the outcome and mediator relationship and all measured variables are binary.

## 1. Introduction

In medicine, biology, economics, or agriculture, it is often of interest to estimate the causal effect of an intervention on an outcome, while removing the effect of a possible mediator. Several such effects have been discussed in the literature; for instance, the controlled direct effect (CDE), in which the mediator is held to a constant level for all subjects, and the natural direct effect (NDE), in which the mediator is held to the “natural” level it would attain, for each subject, had the intervention been set to a particular level. Arguably, the NDE is less intuitive than the CDE. An example that may help to clarify the NDE concerns the direct effect of smoking on lung cancer, where one wishes to set all other comorbidities to the level they would have been for a subject, had the subject not smoked. This NDE is clearly of interest, and may in fact be of greater clinical relevance than the CDE, which in this example requires setting all subjects’ comorbidities to fixed levels—a clearly unrealistic intervention.

Daniel *and others* (2015) and Steen *and others* (2017) introduce estimands with two mediators, discuss estimation of natural direct, controlled direct, and indirect effects, and show the decompositions of the total effect (TE) into the natural effects. However, there are several other direct, and indirect, effect estimands that are crosses of natural and controlled effects that can be considered when there are multiple mediators. We define the cross-NDE and -CDE in the setting of two potentially sequential mediators. No work, to our knowledge, has introduced these estimands previously.

We believe these estimands are of interest in any setting where one might consider setting one mediator to a constant level while setting the other mediator to its natural level under treatment or control. Although we focus on conceptual examples from immunology in vaccination trials in coronavirus disease 2019 (COVID-19), one could imagine that these effects would be of interest in many other scenarios such as political science, sociology, and engineering. For example, crosses of controlled and natural effects might be of interest when considering the direct effect of a law that reduces school funding on graduation rates while setting infrastructure decline to zero and allowing teacher-to-student ratios to be held at the natural level they would be under the new law.

If a mediator is confounded, i.e., has a common cause with the outcome of interest, then conditioning on the mediator opens a noncausal pathway from the intervention to the outcome, even if the intervention is randomized. When these confounders can be controlled, the cross-direct effects can be identified under certain assumptions. We provide a set of assumptions that we prove allow for the identification and point estimation of our proposed cross-direct effects. Identification requires the assumption of no residual or uncontrolled confounders between the mediator and the outcome, which is strong and untestable.

Instead of, or in addition to, providing a point estimate under this strong assumption, nonparametric bounds can provide a range guaranteed to include the unidentifiable causal effect of interest in the presence of uncontrolled confounding. Bounds are potentially informative, i.e., not ranging over the entire parameter space, even in the presence of unknown and uncontrolled confounding. Numeric bounds are often possible to compute regardless of the type of variables, while tight symbolic bounds can be derived using linear programming when all measured variables are binary. Symbolic bounds have the advantage of not requiring rederivation for each new data set as they apply to all data conforming to a particular causal model. Cai *and others* (2007) used the linear programming technique of Balke and Pearl (1994) to derive tight symbolic bounds for the CDE in a setting with one binary mediator and binary outcome and intervention. Sjölander (2009) extended the bounds of Cai *and others* (2007) to the natural effects in the same setting also using linear programming. Gabriel *and others* (2022) derived tight symbolic bounds for a large number of decomposition effects as well as the CDE in the setting of two sequential mediators, where all measured variables are binary using the linear programming method of Sachs *and others* (2022). We use the same method to provide tight symbolic bounds for our proposed cross-direct effects in settings where all measured variables are binary.

The article is organized as follows. In Section 2, we provide our notation and outline our proposed estimands and settings of interest. In Section 3, we discuss some conceptual examples of scenarios where these estimands would be of interest. In Section 4, we provide a set of sufficient assumptions and equations for the identification of our proposed cross-direct effects. In Section 5, we provide the bounds for each of the settings and estimands of interest. In Section 6, we illustrate our estimands and calculate the point estimates and bounds in two data sets: a real data example from a framing experiment conducted in psychology, and a synthetic data example modeled after an mRNA vaccine trial to prevent COVID-19. Finally, in Section 7, we discuss limitations and future areas of research.

## 2. Preliminaries

### 2.1 Notation and settings

Let $X$ and $Y$ be the intervention and outcome of interest, respectively. Let $M_1$ and $M_2$ be two mediators on paths from $X$ to $Y$. Let $\boldsymbol{U}$ be an unmeasured set of confounders for $Y$, $M_1$, and $M_2$, and possibly $\boldsymbol{C}$ that denotes a measured set of confounders between $X$, $Y$, $M_1$, and $M_2$, and possibly $\boldsymbol{U}$.

**Fig. 1.**
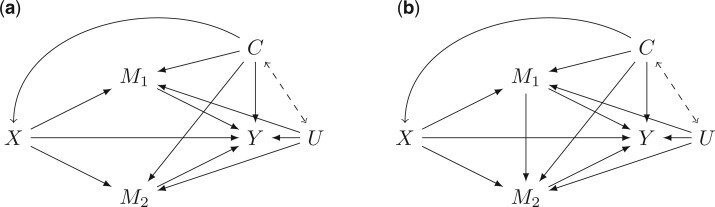
Causal diagrams of the settings of interest. $X$ is the intervention, $M_1,M_2$ the two mediators, and $Y$ the outcome. $C$ and $U$ denote measured and unmeasured confounders, respectively.

The directed acyclic graph (DAG) in Figure 1(a) encodes the following nonparametric structural equation models (NPSEM) (Pearl, 2009):


\begin{eqnarray*}
\label{seqs}
\boldsymbol{c} &=& g_{\boldsymbol{C}}(\boldsymbol{u},\epsilon_{C}),\\
x &=& g_X(\boldsymbol{c}, \epsilon_X)\\
m_1 &=& g_{M_1}(\boldsymbol{u}, x, \boldsymbol{c}, \epsilon_{M_1}) \\
m_2 &=& g_{M_2}(\boldsymbol{u}, x, \boldsymbol{c}, \epsilon_{M_2}) \\
y &=& g_Y(\boldsymbol{u}, m_1, m_2, x, \boldsymbol{c}, \epsilon_Y),
\end{eqnarray*}

for some response functions $g_Y, g_{M_1}, g_{M_2}, g_X,g_{\boldsymbol{C}}$. The unmeasured variables $\boldsymbol{U}$ and the set of $\epsilon$ represent errors due to omitted factors. Given the values of the errors and the values of a variable’s parents in the graph, the value of the variable is determined by its response function. The errors determine the manner in which the variable is determined from its parents, and we note that $Y, M_1, M_2$, and possibility $\boldsymbol{C}$ have a common error variable $\boldsymbol{U}$. In Figure 1(b), we additionally allow for $M_1$ to affect $M_2$, so that the structural equation for $m_2$ is instead:


\begin{eqnarray*}
m_2 &=& g_{M_2}(\boldsymbol{u}, x, m_1,\boldsymbol{c},\epsilon_{M_2});
\end{eqnarray*}

all other equations are the same. The dashed double arrow between $\boldsymbol{C}$ and $\boldsymbol{U}$ in Figure 1 indicates that we allow for a causal relationship between the two sets of confounders but do not define what that relationship is as both directions of the arrow are possible and would not change our results.

### 2.2 Estimands

We let $Y(x)$ denote the potential outcome $Y$ under the intervention that sets $X$ to level $x$, and $Y(x, m_1, m_2)$ be the equivalent under an intervention that sets $X$ to level $x$, $M_1$ to $m_1$, and $M_2$ to $m_2$.

There are several other potential outcomes of relevance in the setting with two mediators, depending on whether there is a causal effect of one of the mediators $M_1$ on the other $M_2$, as in Figure 1(b), or whether such an effect is absent, as in Figure 1(a). Define $Y(x, m_1, M_2(x_2))$ to be the potential outcome $Y$ under an intervention that sets $X$ to $x$, $M_1$ to $m_1$, and $M_2$ to the level it would take on under an intervention setting $X$ to $x_2$; similarly, define $Y(x, M_1(x_1), m_2)$.

When $M_1$ has an effect on $M_2$, as in Figure 1(b), there are novel and additional estimands to consider, based on different potential outcomes. Let $Y(x, m_1, M_2(x_2, m'_1))$ be the potential outcome $Y$ under the intervention that sets $X$ to $x$, $M_1$ to $m_1$ and $M_2$ to the level it would take on under the intervention that sets $X$ to $x_2$ and $M_1$ to $m'_1$. Similarly, let $Y(x, M_1(x_1), M_2(x_2, m_1))$ be the potential outcome $Y$ under the interventions that sets $X$ to $x$, $M_1$ to the level it would take on under the intervention that sets $X$ to $x_1$, and $M_2$ to the level it would take on under the intervention that sets $X$ to $x_2$ and $M_1$ to $m_1$. Finally, let $Y(x, m_1, M_2(x_2, M_1(x_3)))$ be the potential outcome of $Y$ under the intervention that sets $X$ to $x$, $M_1$ to $m_1$ and $M_2$ to the level it would take on under the intervention that sets $X$ to $x_2$ and $M_1$ to the level it would take on had $X$ been set to $x_3$.

When the intervention is randomized or there are no unmeasured confounders between $X$ and $Y$, the total effect of $X$ on $Y$, which we define in counterfactual terms as,


$$
\mbox{TE} = E\{Y(x)\} -E\{Y(x')\}
$$

is identified. We are interested in the direct effect of $X$ on $Y$ setting each mediator to either a constant level or the level they would have taken for each subject had $X$ been set to some level $x$. Daniel *and others* (2015) describe the CDEs and the NDEs for two or more mediators, as well as indirect effects and decompositions of the TE in these settings.

Although this was not discussed in Daniel *and others* (2015), when there are two mediators, one mediator could be held or controlled to a constant level, while the other be set to the level it would take on for each subject if the intervention were set to a constant level. We call these the cross-NDE and CDE. Under Figure 1(a) for two mediators, there are two relevant estimands of this type:


\begin{eqnarray*}
\Delta \!\! E\{Y(x \rightarrow x', M_1(x_1), m_2)\}= E\{Y(x, M_1(x_1), m_2)\} - E\{Y(x', M_1(x_1), m_2)\}
\\ \mbox{ and,} \\
\Delta \!\! E\{Y(x \rightarrow x', m_1, M_2(x_2))\} =E\{Y(x, m_1, M_2(x_2))\} - E\{Y(x', m_1, M_2(x_2)\}\end{eqnarray*}

Under Figure 1(b), there are three additional estimands of this cross-NDE and CDE type that are distinct. Table 1 gives all the effect names and their definitions.

**Table 1.** Effect reference table

DAG	Effect	Definition
1(a) and (b)	$\Delta \!\! E\{Y(x \rightarrow x', M_1(x_1), m_2)\}$	$E\{Y(x, M_1(x_1), m_2)\} -$ $\; E\{Y(x', M_1(x_1), m_2)\}$
1(a) and (b)	$\Delta \!\! E\{Y(x \rightarrow x', m_1, M_2(x_2))\}$	$E\{Y(x, m_1, M_2(x_2))\} - $ $\; E\{Y(x', m_1, M_2(x_2)\}$
1(b)	$\Delta \!\! E\{Y(x \rightarrow x', m_1, M_2(x_2, m_3))\}$	$E\{Y(x, m_1, M_2(x_2, m_3))\} -$ $\; E\{Y(x', m_1, M_2(x_2,m_3))\}$
1(b)	$\Delta \!\! E\{Y(x \rightarrow x', m_1, M_2(x_2, M_1(x_3)))\}$	$E\{Y(x, m_1, M_2(x_2, M_1(x_3)))\}-$ $\;E\{Y(x', m_1, M_2(x_2, M_1(x_3)))\} $
1(b)	$\Delta \!\! E\{Y(x \rightarrow x', M_1(x_1), M_2(x_2,m_1))\}$	$E\{Y(x, M_1(x_1), M_2(x_2,m_1))\}- $ $\;E\{Y(x',M_1(x_1), M_2(x_2,m_1))\}$

In the settings depicted in Figure 1, we will focus on the cross-direct effects introduced above, although similar indirect effects are likely also of interest. One can also see that there are direct extensions of these cross-direct effects in settings with three or more, possibly sequential, mediators.

While these estimands may seem like pure thought experiments, they may be useful for assessing mechanistic pathways and therefore potentially mechanistic surrogate evaluation (Plotkin and Gilbert, 2012). Additionally, although it is not immediately clear how or if these effects are part of a decomposition of the TE or some CDE, we believe they are of interest individually, particularly in immunology.

## 3. Conceptual examples

Goel *and others* (2021) found that 1 week after the second dose of vaccination with an mRNA vaccine (Pfizer BNT162b2 or Moderna mRNA-127) SARS-CoV-2 infection naive subjects had seeming independence of the proportion of memory B-cells that recognize spike protein ($M_1$) and the amount of circulating anti-spike protein IGg antibody ($M_2)$. Thus, Figure 1(a) may be a depiction of this setting.

There are other mechanisms of vaccine protection beyond B-cells and antibody. To better understand the suite of mechanisms, it would be of interest to estimate the direct effect of vaccination by setting one of these mediators to an undetectable level and the other to the natural level that would occur under vaccination, or nonvaccination; for example, the percentage of mediation by antibodies was of interest in Gilbert *and others* (2022). In this case, the estimand $\Delta \!\! E\{Y(1 \rightarrow 0, M_1(1), 0)\}$ may be of interest.

Goel *and others* (2021) also found that baseline memory B-cells prior to vaccine with an mRNA vaccine in SARS-CoV-2 recovered individuals was predictive of circulating antibody levels postvaccination. Although baseline memory B-cell percentage cannot be a mediator, the percentage of B-cells post dose two of mRNA vaccination is likely highly correlated with baseline levels. Thus, Figure 1(b) might be a depiction of the percentage of spike-positive B-cells after the second dose of the vaccine as mediator one, or ($M_1$) in Figure 1(b), and the circulating antispike IGg postdose two of the vaccine as mediator two, or ($M_2$) in Figure 1(b). Therefore, in previously infected individuals, $\Delta \!\! E\{Y(1 \rightarrow 0, 0, M_2(1,M_1(0)))\}$ and $\Delta \!\! E\{Y(1 \rightarrow 0, M_1(1), M_2(1,0))\}$, may be of interest to help tease out the primary route of protection. Although in this example we are technically considering a two-part intervention, if one would considers $X$ as the intervention of receiving two doses, versus no doses or one dose, then Figure 1(b) is a depiction of this setting.

## 4. Sufficient assumptions for identification

All cross-direct effects are nonparametrically identified under our NPSEMs above in the absence of $U$. Under Figure 1(a), the NPSEMs become


\begin{eqnarray*}
\label{seqs2}
\boldsymbol{c} &=& g_{\boldsymbol{C}}(\boldsymbol{\epsilon_C}),\\
x &=& g_X( \boldsymbol{c}, \epsilon_X),\\
m_1 &=& g_{M_1}(\boldsymbol{c},x, \epsilon_{M_1}) \\
m_2 &=& g_{M_2}(\boldsymbol{c},x, \epsilon_{M_2}) \\
y &=& g_Y(\boldsymbol{c},x, m_1, m_2, \epsilon_Y)
\end{eqnarray*}

and under Figure 1(b), the equation for $m_2$ becomes


\begin{eqnarray*}
\label{seqs3}
m_2 &=& g_{M_2}(\boldsymbol{c},x, m_1, \epsilon_{M_2}) \\
\end{eqnarray*}

with all other equations remaining the same. The assumptions based on the NPSEMs imply assumptions MC.1–MC.2 and MCN.3–MCN.5 of Daniel *and others* (2015). In addition to the assumptions encoded by the NPSEM, we make the treatment and mediator positivity assumptions. The assumptions of Daniel *and others* (2015) in terms of our variables that are implied by our NPSEMs and the treatment and mediator positivity assumptions are formally stated in the Supplementary material available at *Biostatistics* online prior to the proof of Proposition 1.

**Proposition 1:** Under the above NPSEMs, we have:


\begin{eqnarray*}
\mbox{a.) }
E\{Y(x, M_1(x_1),m_2)\}&=&\int_{\Omega_{\boldsymbol{C}}} \int_{\Omega_{M_{1}}} \mbox{E}\{Y|\boldsymbol{C}=\boldsymbol{c},X=x, M_1=m_1, M_2=m_2\}\\
&&\times f_{M_{1}|\boldsymbol{C},X}(m_1|\boldsymbol{c},x_1)f_{\boldsymbol{C}}(\boldsymbol{c}){\rm d}m_1{\rm d}\boldsymbol{c}.\end{eqnarray*}

Where $\Omega_{Q}$ denotes the support of a variable, and $\int_{\Omega_{Q}}$ is taken to be $\sum_{\Omega_{Q}}$ when $Q$ is discrete.

By symmetry, when there is no effect of $M_1$ on $M_2$, as in Figure 1(a), one can simply switch $M_1$ and $M_2$, in the above, which is not the case under Figure 1(b). When there is an effect of $M_1$ on $M_2$, the other measures can become important to consider.


\begin{eqnarray*}
\mbox{b.) }
E\{Y(x, m_1,M_2(x_2,m'_1))\} &=&\int_{\Omega_{\boldsymbol{C}}} \int_{\Omega_{M_{2}}} \mbox{E}\{Y|\boldsymbol{C}=\boldsymbol{c},X=x, M_1=m_1, M_2=m_2\}\\
&&\times f_{M_2|\boldsymbol{C},X,M_1}(m_2|\boldsymbol{c},x_2,m'_1)f_{\boldsymbol{C}}(\boldsymbol{c}){\rm d}m_2{\rm d}\boldsymbol{c}.\\
\mbox{c.) }
E\{Y(x, m_1,M_2(x_2,M_1(x_3)))\} &=&\int_{\Omega_{\boldsymbol{C}}}\int_{\Omega_{M_{1}}} \int_{\Omega_{M_{2}}} \mbox{E}\{Y|\boldsymbol{C}=\boldsymbol{c},X=x, M_1=m_1, M_2=m_2\}\\
&&\times f_{M_2|\boldsymbol{C},X,M_1}(m_2|\boldsymbol{c},x_2,m'_1)f_{M_1|\boldsymbol{C},X}(m'_1|\boldsymbol{c},x_3)f_{\boldsymbol{C}}(\boldsymbol{c}){\rm d}m_2{\rm d}m'_1{\rm d}\boldsymbol{c}.\\
\mbox{d.) }
E\{Y(x, M_1(x_1),M_2(x_2, m'_1))\}&=& \int_{\Omega_{\boldsymbol{C}}} \int_{\Omega_{M_{1}}} \int_{\Omega_{M_{2}}} \mbox{E}\{Y|\boldsymbol{C}=\boldsymbol{c},X=x, M_1=m_1, M_2=m_2\}\\
&&\times f_{M_2|\boldsymbol{C},X,M_1}(m_2|\boldsymbol{c},x_2,m'_1)f_{M_1|\boldsymbol{C},X}(m_1|\boldsymbol{c},x_1)f_{\boldsymbol{C}}(\boldsymbol{c}){\rm d}m_2{\rm d}m_1{\rm d}\boldsymbol{c}.
\end{eqnarray*}

With the identification of the expectations, one can identify all of the effects in Table 1.

The proof of Proposition 1 is in Supplementary material available at *Biostatistics* online. We note that one of the assumptions required to prove the above is no residual confounding, which is untestable. Using nonparametric bounds, we can provide a range of possible values when residual confounding prevents the identification of the causal effect of interest.

## 5. Novel bounds for binary data

When there is residual or uncontrolled confounding between the mediators and the outcome, none of the estimands are identified. In settings where $Y$, $X$, $M_1$, and $M_2$ are all categorical with finite domain, we can use the linear programming method implemented in the $\texttt{R}$ package $\texttt{causaloptim}$ to obtain tight nonparametric bounds on the effects in Table 1 (Sachs *and others*, 2022). When variables of interest are all assumed categorical, there exists a canonical partitioning of the unmeasured confounder $U$ into finite states, as described by Sachs *and others* (2022). In this partitioning, when the variables are all binary, the response function corresponding to each variable in the DAG is categorical with $2^{2^k}$ levels, where $k$ is the number of parents of that variable in the DAG. There are thus a finite set of probabilities associated with the joint response function variable distribution for $Y, M_1, M_2 \vert X$: 128 in the setting of Figure 1(a) and 16 384 in the setting of Figure 1(b). The 16 estimable probabilities relate linearly to the response function variable probabilities to form constraints and the mediation effects of interest are the linear objectives that we maximize and minimize symbolically using vertex enumeration, resulting in bounds on the counterfactual probabilities in terms of the estimable data distribution.

These bounds apply within levels of a categorical $\boldsymbol{C}$, where the level of $\boldsymbol{C}$ now defines a new population and can be dropped from the NPSEM and DAG, or marginally if $\boldsymbol{C}$ is the empty set. Either of these scenarios is analogous to a setting where $X$ is randomized, either within levels of $\boldsymbol{C}$ as in stratified randomization, or marginally, as we allow for residual confounding of the mediators and the outcome, but not for residual confounding of these variables and the exposure. Define the shorthand notation for probabilities as:


$$
p_{ym_1m_2{\cdot}x} = p\{Y=y, M_1=m_1, M_2=m_2|X=x\}.
$$

For example, $p_{111{\cdot}1} = p\{Y=1, M_1=1, M_2=1|X=1\}$.

**Result 1:**

The bounds given below are valid and tight for $\Delta \!\! E\{Y(1 \rightarrow 0, M_1(0), 0)\}$ under Figure 1(a) or (b).


\begin{align*}
& \Delta \!\! E\{Y(1 \rightarrow 0, M_1(0), 0)\} \geq \\
& \max\left\{\begin{array}{l}
-1 - p_{100\cdot 0} + p_{010\cdot 0} + p_{110\cdot 1} - p_{001\cdot 0} - p_{101\cdot 0}\\
-2 + 2p_{000\cdot 0} + p_{100\cdot 0} + p_{100\cdot 1} + p_{010\cdot 0} + p_{001\cdot 0} + p_{101\cdot 0}\\
-1 + p_{000\cdot 0} + p_{010\cdot 0}\end{array}\right\},
\end{align*}

and


\begin{align*}
& \Delta \!\! E\{Y(1 \rightarrow 0, M_1(0), 0)\} \leq \\
& \min\left\{\begin{array}{l}
1 + p_{000\cdot 0} - p_{010\cdot 1} - p_{110\cdot 0} + p_{001\cdot 0} + p_{101\cdot 0}\\
1 - p_{100\cdot 0} - p_{110\cdot 0}\\
2 - p_{000\cdot 0} - p_{000\cdot 1} - 2p_{100\cdot 0} - p_{110\cdot 0} - p_{001\cdot 0} - p_{101\cdot 0}\end{array}\right\}.
\end{align*}

Under Figure 1(a), the bounds in Result 1 apply directly to the estimand $\Delta \!\! E\{Y(1 \rightarrow 0, 0, M_2(0))\}$ by symmetry as $M_1$ does not have an effect on $M_2$. This is not the case in Figure 1(b). If there is an effect of $M_1$ on $M_2$, as in Figure 1(b), there are other estimands that may also be important, and that will differ from the above estimands.

**Result 2:**

The bounds given below are valid and tight for $\Delta \!\! E\{Y(1 \rightarrow 0, 0, M_2(0, 0))\}$ under Figure 1(b).


\begin{align*}
&\Delta \!\! E\{Y(1 \rightarrow 0, 0, M_2(0, 0))\} \geq \\
& \max\left\{\begin{array}{l}
-2 + p_{000\cdot 0} + 2p_{001\cdot 0} + p_{101\cdot 0} + p_{101\cdot 1}\\
-2 + 2p_{000\cdot 0} + p_{100\cdot 0} + p_{100\cdot 1} + p_{001\cdot 0}\\
-1 + p_{000\cdot 0} + p_{001\cdot 0}\end{array}\right\},
\end{align*}

and


\begin{align*}
& \Delta \!\! E\{Y(1 \rightarrow 0, 0, M_2(0, 0))\} \leq \\
& \min\left\{\begin{array}{l}
2 - p_{100\cdot 0} - p_{001\cdot 0} - p_{001\cdot 1} - 2p_{101\cdot 0}\\
1 - p_{100\cdot 0} - p_{101\cdot 0}\\
2 - p_{000\cdot 0} - p_{000\cdot 1} - 2p_{100\cdot 0} - p_{101\cdot 0}\end{array}\right\}.
\end{align*}

**Result 3:**

The bounds given below are valid and tight for $\Delta \!\! E\{Y(1 \rightarrow 0, M_1(0), M_2(0,0))\}$ under Figure 1(b).


\begin{align*}
& \Delta \!\! E\{Y(1 \rightarrow 0, M_1(0), M_2(0,0))\} \geq \\
& \max\left\{\begin{array}{l}
-2 + p_{000\cdot 0} + 2p_{001\cdot 0} + p_{101\cdot 0} + p_{101\cdot 1}\\
-2 + 2p_{000\cdot 0} + p_{100\cdot 0} + p_{100\cdot 1} + p_{001\cdot 0}\\
-1 + p_{000\cdot 0} + p_{001\cdot 0}\\
-1 + p_{000\cdot 0} - p_{000\cdot 1} - p_{100\cdot 1} + p_{010\cdot 0} - p_{010\cdot 1} - p_{110\cdot 1} + p_{001\cdot 0} - p_{001\cdot 1} - p_{101\cdot 1} - p_{011\cdot 1}\\
-2 + p_{000\cdot 0} + p_{110\cdot 1} + p_{001\cdot 0} + p_{011\cdot 0}\end{array}\right\},
\end{align*}

and


\begin{align*}
& \Delta \!\! E\{Y(1 \rightarrow 0, M_1(0), M_2(0,0))\} \leq \\
& \min\left\{\begin{array}{l}
2 - p_{100\cdot 0} - p_{001\cdot 0} - p_{001\cdot 1} - 2p_{101\cdot 0}\\
2 - p_{100\cdot 0} - p_{110\cdot 0} - p_{101\cdot 0} - p_{011\cdot 1}\\
1 + p_{000\cdot 0} + p_{010\cdot 0} - p_{010\cdot 1} + p_{110\cdot 0} + p_{001\cdot 0} + p_{011\cdot 0}\\
1 - p_{100\cdot 0} - p_{101\cdot 0}\\
2 - p_{000\cdot 0} - p_{000\cdot 1} - 2p_{100\cdot 0} - p_{101\cdot 0}\end{array}\right\}.
\end{align*}

**Result 4:**

The bounds given below are valid and tight for $\Delta \!\! E\{Y(1 \rightarrow 0, 0, M_2(0,M_1(0)))\}$ under Figure 1(b).


\begin{align*}
& \Delta \!\! E\{Y(1 \rightarrow 0, 0, M_2(0,M_1(0)))\} \geq \\
& \max\left\{\begin{array}{l}
-1 - p_{100\cdot 0} - p_{010\cdot 0} - p_{110\cdot 0} + p_{001\cdot 0} + p_{101\cdot 1}\\
-2 + 2p_{000\cdot 0} + p_{100\cdot 0} + p_{100\cdot 1} + p_{010\cdot 0} + p_{110\cdot 0} + p_{001\cdot 0}\\
-1 + p_{000\cdot 0} + p_{001\cdot 0}\end{array}\right\},
\end{align*}

and


\begin{align*}
& \Delta \!\! E\{Y(1 \rightarrow 0, 0, M_2(0,M_1(0)))\} \leq \\
& \min\left\{\begin{array}{l}
1 + p_{000\cdot 0} + p_{010\cdot 0} + p_{110\cdot 0} - p_{001\cdot 1} - p_{101\cdot 0}\\
1 - p_{100\cdot 0} - p_{101\cdot 0}\\
2 - p_{000\cdot 0} - p_{000\cdot 1} - 2p_{100\cdot 0} - p_{010\cdot 0} - p_{110\cdot 0} - p_{101\cdot 0}\end{array}\right\}.
\end{align*}

The bounds corresponding to the other levels of $\{x_1,x_2,x_3\}$ are given in the supplementary materials; results are labeled based on the estimand, 1, 2, 3, or 4, and then b to h.

## 6. Data examples

We illustrate our novel estimands using two data sets. The first is a real data example, the framing data from the $\texttt{mediation}$ package in R (Tingley *and others*, 2014). The framing data contains 265 rows and 15 columns of data from a framing experiment conducted by Brader *and others* (2008). The experiment was to expose subjects to a news story that is randomized to a tone, positive or negative, and to whether the news story features a Latino or European immigrant. We consider the binary intervention that is 1 if the news story features a Latino immigrant with a negative tone and 0 otherwise (either positive tone or European immigrant), as this was the treatment considered in the original study. These data include covariates and have an ordinal outcome and mediators and we use the outcome of the four-point scale measuring a subject’s attitude toward increased immigration, with larger values indicating more negative attitudes, by dichotomizing into 1 or 2 as low, and 3 or 4 as high. Similarly for the mediators, a subject’s negative feeling during the experiment, which was originally measured on the numeric scale ranging between 3 and 12, we dichotomized this as low, below 8 and high above or equal to 8, and a subject’s perceived harm caused by increased immigration. This perceived harm was originally on a numeric scale between 2 and 8, which we dichotomize to zero for less than 6, and one for 6 or greater. We consider the dichotomized emotional state as $M_1$ which may causally impact the perceived harm, $M_2$. The additional baseline covariates in the data set we consider are age, the highest level of education, gender, and income.

For point estimation of the effects of interest, we assumed that the measured covariates are the only confounders. We then specified logistic regression models for $Y | \boldsymbol{C}, X, M_1, M_2$, $M_1 | \boldsymbol{C}, X$, and $M_2 | \boldsymbol{C}, X, M_1$ that include all covariates and all two-way interactions among the covariates, treatment, and mediators. We estimated $E\{Y(x, m_1,M_2(x_2,m'_1))\}$ as


\begin{eqnarray*}
&& \hat{E}\{Y(x, m_1,M_2(x_2,m'_1))\}\\
&&\quad{} = n^{-1}\sum_{i = 1}^n \sum_{j \in \{0,1\}} \hat{p}\{Y = 1 | \boldsymbol{C} = \boldsymbol{C_i}, X = x, M_{1} = m_1, M_{2} = j\}\\
&&\qquad{} \times \hat{p}\{M_2 = j | \boldsymbol{C} = \boldsymbol{C_i}, X = x_2, M_1 = m'_1\}.
\end{eqnarray*}

In this expression, the estimated probabilities were obtained from the logistic regression models, and the population distribution of $\boldsymbol{C}$ is estimated with its sample distribution. All other terms of our estimands were estimated analogously. For inference, we use the nonparametric bootstrap. Although our estimation procedure can be followed directly when the exposure, outcome, and mediators are binary, we believe that estimation in other settings that are identified can directly follow that suggested in Daniel *and others* (2015).

**Fig. 2.**
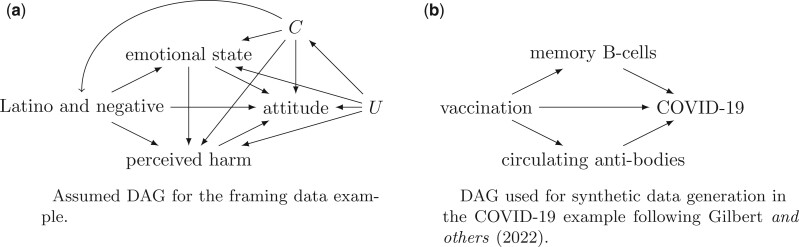
DAGs for real and synthetic data examples. In Example 1, we assume the DAG in the left panel, in Example 2, we generate data that fit this DAG and also the summary statistics of Gilbert *and others* (2022).

While some of the confidence intervals exclude the null value of 0, these estimates rely on the untestable assumptions of no residual confounding and correct specification of the models. In the calculation of the bounds, we ignore the measured confounders, thus they effectively become part of the set of unmeasured confounders. The bounds do not rely on the assumption of no residual confounding, and although all bounds cover zero in this example, the width of the bounds and the causal effects they cover differ greatly over the estimands. As can be seen in Figure 2, $\Delta \!\! E\{Y(1 \rightarrow 0, M_1(1), 1)\}$, which is the same under either Figure 1(a) or (b), have mostly positive values, implying mostly a range of harm or increased risk of negative attitudes. This suggests that a negatively toned news article about a Latino immigrant is most likely to cause negative attitudes, although no change cannot be ruled out, if subjects have a high emotional state and the perceived harm they would have had if they had been exposed to the negatively toned article about a Latino. Compared with the estimand $\Delta \!\! E\{Y(1 \rightarrow 0, M_1(1), M_2(1,0))\}$ which has a smaller point estimate near 0, and wider bounds ranging from around $-0.75$ to $0.9$, we can infer little about the cause of a negatively toned news article about a Latino immigrant controlling the emotional state at 0 in its effect on perceived harm, but otherwise having emotional state and perceived harm at their natural levels under treatment.

The second data example is a synthetic data set constructed to roughly follow the data used in Gilbert *and others* (2022), who analyzed the effect of circulating antibody at day 57 postfirst dose of the Moderna mRNA vaccine on the disease risk and vaccine efficacy (VE) among those who received two doses. We generate the vaccination variable as $X$ (0 if no doses and 1 if two doses) as Bernoulli with probability 0.5, a continuous memory B-cell response ($M_1$) as Gaussian with mean $0.5 + 1.0 X$ and variance 1 that would have been measured at day 57 after vaccination. The day 57 circulating antibody $M_2$ is generated as $\exp(Y)$ where $Y$ is Gaussian with mean $6.5 + 0.5 X$ and standard deviation 0.3. Based on the B-cell and antibody responses, we generate a disease indicator as Bernoulli with probability $0.1\times \{\exp(-0.87/900) M_2 - 0.03 M_1 - 0.2\}$ under vaccination and probability $0.1$ under placebo. Informally, one can think of the direct effect of vaccination as primarily due to unmeasured T-cells at day 57. With these parameters, the overall VE is $0.92= 1 - E\{ Y(1) \}/E\{Y(0)\} $ consistent with the VE reported in Gilbert *and others* (2022).

**Fig. 3.**
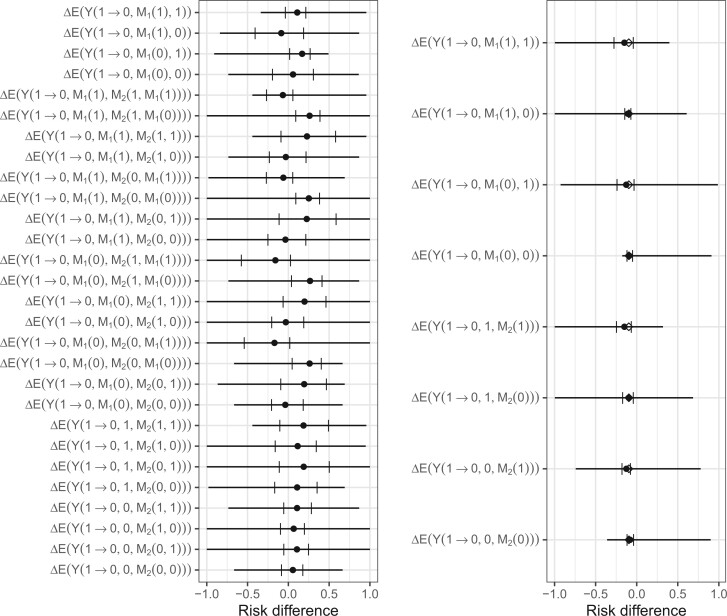
Point estimates (dots) and bootstrap 95$\%$ confidence intervals (left and right brackets) under the assumption of no residual confounding and computed bounds (line ranges) for the different effects using the framing data (left panel) and the synthetic COVID-19 data (right panel) ignoring measured confounders. The diamonds on the covid figure are the estimates based on a sample size of 10 000 000.

We simulated a single trial of 1000 volunteers that had 3 and 51 events in the vaccine and placebo arms, respectively. To compute the estimates and the bounds, we dichotomized the mediators at $M_1=1$ and $M_2=1000$. Figure 3 shows the point estimates with confidence intervals, the true parameter values, and bounds. All point estimates are less than zero, indicating a benefit of vaccination and the 95$\%$ confidence intervals all exclude zero. We do not generate or consider measured confounders in this example for the simplicity of illustration of the bounds. For this reason, the point estimates are computed using the same expressions but with $\boldsymbol{C}$ as the empty set. Note that the bounds do not collapse because they allow for residual confounding and cannot rule out their existence based on the observed probabilities alone.

The estimands allow consideration of the different effects induced by vaccination. For example, $\Delta \!\! E\{ Y(1\rightarrow 0, M_1(0), 0))\}$ represents the direct effect of vaccination while setting antibody $M_2$ to 0 and memory B-cells to $M_1(0)$, reflecting their natural distribution prior to vaccination. The point estimate indicates that the direct effect of vaccination alone, presumably via T-cells, have a benefit in preventing COVID-19. If this effect were large, it would indicate antibody and memory B-cells are not the only things necessary for protection. “T-cell”-based vaccines are showing promise for HIV Hansen *and others* (2019), under which it is believed that the primary route of protection is via T-cell activity.

Although the estimators used in the real and synthetic data examples are not the main focus of this work, we have run some simulations of the estimands both with and without residual confounding. The results of these simulations are presented in the simulation section of Supplementary material available at *Biostatistics* online. The results are as expected, the estimators used are unbiased and the bootstrap confidence intervals have nominal coverage when there is no residual confounding, and the bias is increased and the coverage decreased when residual confounding is present.

## 7. Discussion

We present several novel direct effects that are likely of interest in immunology when there are two, potentially sequential, mediators. We show that the same set of sufficient assumptions for identification given in Daniel *and others* (2015) are sufficient for these cross-NDE and CDE. When these assumptions do not hold due to residual confounding, we provide a means of bounding these estimands when all measured variables are binary.

The formal definitions of the estimands are novel, to our knowledge, but the estimands may have been considered informally in previous immunological settings due to their clear alignment with the goals of understanding mechanisms. To better understand mechanisms of protection, vaccinologists conduct designed or natural experiments where different aspects of the vaccine-induced immune response are manipulated either by removal or insertion. For example, Alter *and others* (2020) harvested vaccine-induced antibodies from vaccinated macaques, infused the antibodies into naïe macaques and demonstrated protection from challenge solely via antibody. A natural experiment evaluated the effect of SARS-CoV-2 vaccination in humans with neuroimmunologic disorders who were on a treatment that removed B-cells. Nonetheless a strong T-cell response was invoked (Kornek *and others*, 2022). Finally, a designed experiment depleted T-cells from vaccinated macaques and showed poorer control of virus following challenge, thus demonstrating the importance of T-cell in protection (Metzner *and others*, 2000). The estimands outlined in this article are an attempt to formalize the targets of such experiments, but in randomized vaccine phase 3 clinical trials in humans where direct manipulation of the direct effects and mediators is not possible. These investigations have likely been undertaken without consideration of how or if the estimands are identifiable. We hope that our work provides a means for further investigation of these estimands, and their use, particularly in the settings of vaccine immunology.

This article is limited in a few ways: in not providing a formal inference procedure for the bounds, and in only providing bounds for settings where all variables are binary. Although we believe estimation of the bounds in the unidentified settings to be straightforward we do not further discuss inference. However, we suggest the inference about the bounds can be obtained using the bootstrap procedure suggested and investigated in Gabriel *and others* (2020). Extensive empirical results were provided showing nominal coverage of quantile bootstrap confidence intervals for similarly constructed bounds. However, it should be noted that standard bootstrap inference is only valid when one can *a priori* rule out being close to the boundary of the parameter space, which for the bounds is not only defined by the natural boundaries $[-1,1]$, but also the true value of the causal effect. Andrews (2000) shows that the standard nonparametric bootstrap is inconsistent near the boundary and provides alternatives to the nonparametric bootstrap.

The restriction to settings with all binary measured variables limits the settings the bounds will cover. However, as demonstrated in the data example, dichotomization can provide one means of using the bounds provided in almost all settings. The information loss associated with dichotomization in the setting of nonparametric bounds is not well studied and may in some settings be large. As the information loss due to residual confounding can also be vast, the balance between these two forms of loss must be considered when using the bounds. Our method of bounds deviation can theoretically allow for all variables to have arbitrarily many levels; deriving these bounds for multilevel categorical settings is future work for the authors and may result in better performance than dichotomization.

An important extension of our work would be to consider intermediate confounders, i.e., confounders for the mediators and the outcome that are affected by the exposure. It has been shown that controlled direct effects are nonparametrically identified in the presence of intermediate confounders, provided that all confounders are measured (Robins, 1999; Goetgeluk *and others*, 2008). However, it has also been shown that natural direct and indirect effects are generally not identifiable, even if all confounders are measured, except under strong parametric assumptions (Avin *and others*, 2005; Tchetgen Tchetgen and VanderWeele, 2014). It would thus be important to determine which, if any, of the cross-effect estimands that we have proposed that are identifiable in the presence of measured intermediate confounder, and to derive bounds for those that are not. This task is complicated by the fact that, in settings with two mediators, there may be different types of intermediate confounders, e.g., those that only confound the first mediator, those that only confound the second mediator, and combinations of these. Furthermore, in addition to the measured intermediate confounders, there may be unmeasured confounders that are not affected by the exposure, which compounds the identifiability problem. Finally, it is not obvious that the linear programming method can be used to derive bounds in the presence of intermediate confounders. We plan to address these complications in future work.

## Supplementary Material

kxac037_Supplementary_Data

## Software

Software in the form of R scripts and complete documentation is available at https://github.com/sachsmc/liger.
