# Supplementary material for: Cross-direct effects in settings with two mediators
Source: Biostatistics. 2022 Sep 2;24(4):1017–30. doi: 10.1093/biostatistics/kxac037 (PMC10583720; doi:10.1093/biostatistics/kxac037)
Supplement: kxac037_Supplementary_Data [file kxac037_supplementary_data.pdf]

Supplementary Materials: "Cross Direct Effects in Settings with Two Mediators"

1 Proof of Proposition 1

We restate the assumptions of Daniel et al. [2015], that align with our DAGs and NPSEMs.

1. Global consistency: (MC.1 and MN.3 of Daniel et al. [2015]) Consistency of (X, M_1, M_2) on Y and consistency of X on M_1, and X, M_1 on M_2,
   $Y_i(x, m_1, m_2) = Y_i | X_i = x, M_{i1} = m_1, M_{i2} = m_2$
   $M_{i1}(x) = M_{i1} | X_i = x$
   $M_{i2}(x, m_1) = M_{i2} | X_i = x, M_{i1} = m_1$

2. Global no unmeasured confounding (MC.2 and MN.4 of Daniel et al. [2015]) No unmeasured confounding of the $(\boldsymbol{C}, M_1, M_2)$-$Y$ relationship, implying the following conditional independencies,
   $Y(x, m_1, m_2) \perp (X, M_1, M_2) | \boldsymbol{C}$
   as well as no unmeasured confounding of the $x - M_1$ or $(X, M_1) - M_2$ relationships, implying the following conditional independencies,
   $M_1(x) \perp X | \boldsymbol{C} = \boldsymbol{c}$,
   $M_2(x, m_1) \perp (M_1, X) | \boldsymbol{C} = \boldsymbol{c}$

3. no measured confounding of (M_1, M_2)-Y that is affected by X, (MN.5 of Daniel et al. [2015]), implying the conditional independencies,
   $M_2(x, m_1) \perp (M_1(x), M_1(x')) | \boldsymbol{C} = \boldsymbol{c}$
   $Y(x, m_1, m_2) \perp (M_2(x, m_1'), M_1(x), M_1(x')) | \boldsymbol{C} = \boldsymbol{c}$.

We further explicitly state the positivity assumptions, for the treatment X and mediators M_1 and M_2, which were clearly used by, but not explicitly stated in Daniel et al. [2015]. These assumptions are as follows:
$p(X = x | C = c) > 0 \ \forall x \in X$ and $c \in C$
and
$p(M_1 = m_1, M_2 = m_2 | C = c, X = x) \ \forall x \in X$, $c \in C$, $m_1 \in M_1$ and $m_2 \in M_2$

The assumptions above and the implied conditional independencies follow from the NPSEM for Figure 1a:

$$
\begin{aligned}
\boldsymbol{c} &= g_{\boldsymbol{C}}(\epsilon_{\boldsymbol{C}}) \\
m_1 &= g_{M_1}(\boldsymbol{c}, x, \epsilon_{M_1}) \\
m_2 &= g_{M_2}(\boldsymbol{c}, x, \epsilon_{M_2}) \\
x &= g_X(\boldsymbol{c}, \epsilon_X), \\
y &= g_Y(\boldsymbol{c}, x, m_1, m_2, \epsilon_Y).
\end{aligned}
$$

Part (a) of Proposition 1 is proven as follows:

$$
\begin{aligned}
& E\{Y(x, M_1(x_1), m_2)\} = \int_{\Omega_{\boldsymbol{C}}} E\{Y(x, M_1(x_1), m_2)|\boldsymbol{C} = \boldsymbol{c}\} f_{\boldsymbol{C}}(\boldsymbol{c}) d\boldsymbol{c} \\
= & \int_{\Omega_{\boldsymbol{C}}} \int_{\Omega_{M_1}} E\{Y(x, m_1, m_2)|M_1(x_1) = m_1, \boldsymbol{C} = \boldsymbol{c}\} f_{M_1(x_1)|\boldsymbol{C}}(m_1|\boldsymbol{c}) f_{\boldsymbol{C}}(\boldsymbol{c}) dm_1 d\boldsymbol{c} \\
= & \int_{\Omega_{\boldsymbol{C}}} \int_{\Omega_{M_1}} E\{Y(x, m_1, m_2)|\boldsymbol{C} = \boldsymbol{c}\} f_{M_1(x_1)|\boldsymbol{C}}(m_1|\boldsymbol{c}) f_{\boldsymbol{C}}(\boldsymbol{c}) dm_1 d\boldsymbol{c} \\
= & \int_{\Omega_{\boldsymbol{C}}} \int_{\Omega_{M_1}} \mathrm{E}\{Y(x, m_1, m_2)|\boldsymbol{C} = \boldsymbol{c}, X = x, M_1 = m_1, M_2 = m_2\} f_{M_1|\boldsymbol{C},X}(m_1|\boldsymbol{c}, x_1) f_{\boldsymbol{C}}(\boldsymbol{c}) dm_1 d\boldsymbol{c} \\
= & \int_{\Omega_{\boldsymbol{C}}} \int_{\Omega_{M_1}} \mathrm{E}\{Y|\boldsymbol{C} = \boldsymbol{c}, X = x, M_1 = m_1, M_2 = m_2\} f_{M_1|\boldsymbol{C},X}(m_1|\boldsymbol{c}, x_1) f_{\boldsymbol{C}}(\boldsymbol{c}) dm_1 d\boldsymbol{c}
\end{aligned}
$$

Where equalities one and two are by the law of total expectation, and equality three from assumption 3. Equality four follows from assumption 2 and 1, and equality five from assumption 1.
By symmetry, when there is no effect of M_1 on M_2, one can simply switch the M_1 and M_2, in the above.
The assumptions above and the implied conditional independencies follow from the NPSEM for Figure 1b:

$$
\begin{aligned}
\boldsymbol{c} &= g_{\boldsymbol{C}}(\epsilon_{\boldsymbol{C}}) \\
m_1 &= g_{M_1}(\boldsymbol{c}, x, \epsilon_{M_1}) \\
m_2 &= g_{M_2}(\boldsymbol{c}, x, m_1, \epsilon_{M_2}) \\
x &= g_X(\boldsymbol{c}, \epsilon_X), \\
y &= g_Y(\boldsymbol{c}, x, m_1, m_2, \epsilon_Y).
\end{aligned}
$$

This holds if all conditional probabilities used are not zero, and thus positivity is needed for this proof to hold.

Part (b) of Proposition 1 is proven as follows:

$$
\begin{aligned}
& E\{Y(x, m_1, M_2(x_2, m_1'))\} = \int_{\Omega_{\boldsymbol{C}}} E\{Y(x, m_1, M_2(x_2, m_1'))|\boldsymbol{C} = \boldsymbol{c}\} f_{\boldsymbol{C}}(\boldsymbol{c}) d\boldsymbol{c} \\
= & \int_{\Omega_{\boldsymbol{C}}} \int_{\Omega_{M_2}} E\{Y(x, m_1, m_2)|M_2(x_2, m_1') = m_2, \boldsymbol{C} = \boldsymbol{c}\} f_{M_2(x_2, m_1')|\boldsymbol{C}}(m_2|\boldsymbol{c}) f_{\boldsymbol{C}}(\boldsymbol{c}) dm_2 d\boldsymbol{c} \\
= & \int_{\Omega_{\boldsymbol{C}}} \int_{\Omega_{M_2}} E\{Y(x, m_1, m_2)|\boldsymbol{C} = \boldsymbol{c}\} f_{M_2(x_2, m_1')|\boldsymbol{C}}(m_2|\boldsymbol{c}) f_{\boldsymbol{C}}(\boldsymbol{c}) dm_2 d\boldsymbol{c} \\
= & \int_{\Omega_{\boldsymbol{C}}} \int_{\Omega_{M_2}} \mathrm{E}\{Y(x, m_1, m_2)|\boldsymbol{C} = \boldsymbol{c}, X = x, M_1 = m_1, M_2 = m_2\} \\
\times & \; f_{M_2|\boldsymbol{C},X,M_1}(m_2|\boldsymbol{c}, x_2, m_1') f_{\boldsymbol{C}}(\boldsymbol{c}) dm_2 d\boldsymbol{c} \\
= & \int_{\Omega_{\boldsymbol{C}}} \int_{\Omega_{M_2}} \mathrm{E}\{Y|\boldsymbol{C} = \boldsymbol{c}, X = x, M_1 = m_1, M_2 = m_2\} f_{M_2|\boldsymbol{C},X,M_1}(m_2|\boldsymbol{c}, x_2, m_1') f_{\boldsymbol{C}}(\boldsymbol{c}) dm_2 d\boldsymbol{c}
\end{aligned}
$$

Where equalities one and two are by the law of total expectation, and equality three from assumption 3. Equality four follows from assumption 2 and 1, and equality five from assumption 1. Again, this holds if all conditional probabilities used are not zero, and thus positivity is needed for this proof to hold.
Part (c) of Proposition 1 is proven as follows:

$$
\begin{aligned}
E\{Y(x, m_1, M_2(x_2, M_1(x_3)))\} &= \int_{\Omega_{\boldsymbol{C}}} E\{Y(x, m_1, M_2(x_2, M_1(x_3)))|\boldsymbol{C} = \boldsymbol{c}\} f_{\boldsymbol{C}}(\boldsymbol{c}) d\boldsymbol{c} \\
= & \int_{\Omega_{\boldsymbol{C}}} \int_{\Omega_{M_1}} \int_{\Omega_{M_2}} E\{Y(x, m_1, m_2)|M_2(x_2, M_1(x_3)) = m_2, M_1(x_3) = m_1', \boldsymbol{C} = \boldsymbol{c}\} \\
\times & f_{M_2(x_2, M_1(x_3))|\boldsymbol{C}, M_1(x_3)}(m_2|\boldsymbol{c}, m_1') f_{M_1(x_3)|\boldsymbol{C}}(m_1'|\boldsymbol{c}) f_{\boldsymbol{C}}(\boldsymbol{c}) dm_2 dm_1' d\boldsymbol{c} \\
= & \int_{\Omega_{\boldsymbol{C}}} \int_{\Omega_{M_1}} \int_{\Omega_{M_2}} E\{Y(x, m_1, m_2)|\boldsymbol{C} = \boldsymbol{c}\} f_{M_2(x_2, M_1(x_3))|\boldsymbol{C}, M_1(x_3)}(m_2|\boldsymbol{c}, m_1') \\
\times & f_{M_1(x_3)|\boldsymbol{C}}(m_1'|\boldsymbol{c}) f_{\boldsymbol{C}}(\boldsymbol{c}) dm_2 dm_1' d\boldsymbol{c} \\
= & \int_{\Omega_{\boldsymbol{C}}} \int_{\Omega_{M_1}} \int_{\Omega_{M_2}} E\{Y(x, m_1, m_2)|\boldsymbol{C} = \boldsymbol{c}, X = x, M_1 = m_1, M_2 = m_2\} f_{M_2|\boldsymbol{C}, M_1, X}(m_2|\boldsymbol{c}, x_2, m_1') \\
\times & f_{M_1|\boldsymbol{C}, X}(m_1'|\boldsymbol{c}, x_3) f_{\boldsymbol{C}}(\boldsymbol{c}) dm_2 dm_1' d\boldsymbol{c} \\
= & \int_{\Omega_{\boldsymbol{C}}} \int_{\Omega_{M_1}} \int_{\Omega_{M_2}} \mathrm{E}\{Y|\boldsymbol{C} = \boldsymbol{c}, X = x, M_1 = m_1, M_2 = m_2\} f_{M_2|\boldsymbol{C}, M_1, X}(m_2|\boldsymbol{c}, x_2, m_1') \\
\times & f_{M_1|\boldsymbol{C}, X}(m_1'|\boldsymbol{c}, x_3) f_{\boldsymbol{C}}(\boldsymbol{c}) dm_2 dm_1' d\boldsymbol{c}
\end{aligned}
$$

Where equalities one and two are by the law of total expectation, and equality three from assumption 3. Equality four follows from assumption 2 and 1, and equality five from assumption 1. This holds if all conditional probabilities used are not zero, and thus positivity is needed for this proof to hold.
Part (d) of Proposition 1 is proven as follows:

$$
\begin{aligned}
E\{Y(x, M_1(x_1), M_2(x_2, m_1'))\} &= \int_{\Omega_{\boldsymbol{C}}} E\{Y(x, M_1(x_1), M_2(x_2, m_1'))|\boldsymbol{C} = \boldsymbol{c}\} f_{\boldsymbol{C}}(\boldsymbol{c}) d\boldsymbol{c} \\
= & \int_{\Omega_{\boldsymbol{C}}} \int_{\Omega_{M_1}} \int_{\Omega_{M_2}} E\{Y(x, m_1, m_2)|M_1(x_1) = m_1, M_2(x_2, m_1') = m_2, \boldsymbol{C} = \boldsymbol{c}\} \\
\times & f_{M_2(x_2, m_1')|\boldsymbol{C}, M_1(x_1)}(m_2|\boldsymbol{c}, m_1) f_{M_1(x_1)|\boldsymbol{C}}(m_1|\boldsymbol{c}) f_{\boldsymbol{C}}(\boldsymbol{c}) dm_2 dm_1 d\boldsymbol{c} \\
= & \int_{\Omega_{\boldsymbol{C}}} \int_{\Omega_{M_1}} \int_{\Omega_{M_2}} E\{Y(x, m_1, m_2)|\boldsymbol{C} = \boldsymbol{c}\} \\
\times & f_{M_2(x_2, m_1')|\boldsymbol{c})}(m_2|\boldsymbol{c}) f_{M_1(x_1)|\boldsymbol{C}}(m_1|\boldsymbol{c}) f_{\boldsymbol{C}}(\boldsymbol{c}) dm_2 dm_1 d\boldsymbol{c} \\
= & \int_{\Omega_{\boldsymbol{C}}} \int_{\Omega_{M_1}} \int_{\Omega_{M_2}} \mathrm{E}\{Y(x, m_1, m_2)|\boldsymbol{C} = \boldsymbol{c}, X = x, M_1 = m_1, M_2 = m_2\} \\
\times & f_{M_2|\boldsymbol{C}, X, M_1}(m_2|\boldsymbol{c}, x_2, m_1') f_{M_1|\boldsymbol{C}, X}(m_1|\boldsymbol{c}, x_1) f_{\boldsymbol{C}}(\boldsymbol{c}) dm_2 dm_1 d\boldsymbol{c} \\
= & \int_{\Omega_{\boldsymbol{C}}} \int_{\Omega_{M_1}} \int_{\Omega_{M_2}} \mathrm{E}\{Y|\boldsymbol{C} = \boldsymbol{c}, X = x, M_1 = m_1, M_2 = m_2\} \\
\times & f_{M_2|\boldsymbol{C}, X, M_1}(m_2|\boldsymbol{c}, x_2, m_1') f_{M_1|\boldsymbol{C}, X}(m_1|\boldsymbol{c}, x_1) f_{\boldsymbol{C}}(\boldsymbol{c}) dm_2 dm_1 d\boldsymbol{c}
\end{aligned}
$$

Where equalities one and two are by the law of total expectation, and equality three from assumption 3. Equality four follows from assumption 2 and 1, and equality five from assumption 1. This holds if all conditional probabilities used are not zero, and thus positivity is needed for this proof to hold.

■

2 Simulations

We generate data as follows. For $i = 1, \ldots, n$, U_i is distributed Bernoulli with probability 0.35 and X_i Bernoulli with probability 0.5. Then we generate M_{1i} as Bernoulli with probability $0.4 - 0.14X_i + \beta_1 U_i$, M_{2i} as Bernoulli with probability $0.3 + 0.25X_i + 0.15M_{1i} + \beta_2 U_i$, and Y_i Bernoulli with probability $p_i + \gamma U_i$, where $p_i = 0.125 + 0.15M_{1i} + 0.2M_{2i}$ if $X_i = 1$ and 0.75 if $X_i = 0$. We consider two scenarios: confounding where $(\beta_1, \beta_2, \gamma) = (-0.1, -0.15, -0.1)$ and no confounding where $(\beta_1, \beta_2, \gamma) = (0, 0, 0)$. We consider 3 sample sizes, $n = 50, 100, 200$ and two of the effects of interest delta2 $= \Delta\mathbb{E}\{Y(1 \to 0, M_1(0), 1)\}$ and delta2 $= \Delta\mathbb{E}\{Y(1 \to 0, 0, M_2(1, M_1(1)))\}$.

For each of 500 simulated replicates, we compute the estimates, percentile based bootstrap confidence intervals, and the bounds. In the table below we report the mean percent bias which is 100 times the difference between the estimate and the true value divided by the true value, the standard deviation of the bias (not on the percent scale), the percent coverage of the bootstrap confidence intervals, the mean lower bound, the mean upper bound, and the mean width of the bounds.

setting	n	%bias	sdbias	coverage	mean LB	meanUB	meanBW
confounding delta1	50	1.565	0.237	0.877	-0.918	0.765	1.684
confounding delta1	100	4.872	0.162	0.938	-0.922	0.773	1.695
confounding delta1	200	0.833	0.104	0.960	-0.917	0.777	1.693
confounding delta2	50	-0.388	0.175	0.881	-0.851	0.370	1.221
confounding delta2	100	-0.272	0.118	0.928	-0.852	0.374	1.227
confounding delta2	200	1.747	0.082	0.946	-0.859	0.368	1.227
no confounding delta1	50	-5.895	0.217	0.895	-0.908	0.720	1.628
no confounding delta1	100	1.918	0.151	0.922	-0.914	0.725	1.639
no confounding delta1	200	-0.297	0.094	0.940	-0.909	0.728	1.637
no confounding delta2	50	-2.898	0.182	0.892	-0.827	0.436	1.264
no confounding delta2	100	0.416	0.124	0.932	-0.827	0.429	1.256
no confounding delta2	200	-0.393	0.079	0.954	-0.823	0.432	1.255

Table 1: Simulation results:

3 Additional bounds

Further bounds for the $\Delta E\{Y(1 \to 0, M_1(x_1), m_2)\}$, of results 1 in the main text for $\Delta E\{Y(1 \to 0, M_1(0), 0)\}$.

Result 1b:

The bounds given below are valid and tight for $\Delta E\{Y(1 \to 0, M_1(0), 1)\} = E\{Y(1, M_1(0), 1)\} - E\{Y(0, M_1(0), 1)\}$ under Figure 1a.

$$\Delta E\{Y(1 \to 0, M_1(0), 1)\} \geq$$
$$\max \left\{ \begin{array}{l} -p_{000\cdot 0} - p_{000\cdot 1} - p_{100\cdot 0} - p_{100\cdot 1} - p_{010\cdot 1} - p_{110\cdot 1} - p_{001\cdot 1} - p_{101\cdot 0} - p_{101\cdot 1} + p_{011\cdot 0} - p_{011\cdot 1} \\ -2 + p_{000\cdot 0} + p_{100\cdot 0} + 2p_{001\cdot 0} + p_{101\cdot 0} + p_{101\cdot 1} + p_{011\cdot 0} \\ -1 + p_{001\cdot 0} + p_{011\cdot 0} \end{array} \right\},$$

and

$$\Delta E\{Y(1 \to 0, M_1(0), 1)\} \leq$$
$$\min \left\{ \begin{array}{l} 2p_{000\cdot 0} + 2p_{100\cdot 0} + p_{010\cdot 0} + p_{110\cdot 0} + 2p_{001\cdot 0} + p_{101\cdot 0} + p_{011\cdot 0} - p_{011\cdot 1} \\ p_{000\cdot 0} + p_{100\cdot 0} + p_{010\cdot 0} + p_{110\cdot 0} + p_{001\cdot 0} + p_{011\cdot 0} \\ 1 + p_{010\cdot 0} + p_{110\cdot 0} - p_{001\cdot 1} - p_{101\cdot 0} + p_{011\cdot 0} \end{array} \right\}.$$

Result 1c:

The bounds given below are valid and tight for $\Delta E\{Y(1 \to 0, M_1(1), 0)\} = E\{Y(1, M_1(1), 0)\} - E\{Y(0, M_1(1), 0)\}$ under Figure 1a.

$$\Delta E\{Y(1 \to 0, M_1(1), 0)\} \geq$$
$$\max \left\{ \begin{array}{l} -1 - p_{000\cdot 1} + p_{010\cdot 0} + p_{110\cdot 1} - p_{001\cdot 1} - p_{101\cdot 1} \\ -2 + p_{000\cdot 0} + p_{000\cdot 1} + 2p_{100\cdot 1} + p_{110\cdot 1} + p_{001\cdot 1} + p_{101\cdot 1} \\ -1 + p_{100\cdot 1} + p_{110\cdot 1} \end{array} \right\},$$

and

$$\Delta E\{Y(1 \to 0, M_1(1), 0)\} \leq$$
$$\min \left\{ \begin{array}{l} 1 + p_{100\cdot 1} - p_{010\cdot 1} - p_{110\cdot 0} + p_{001\cdot 1} + p_{101\cdot 1} \\ 1 - p_{000\cdot 1} - p_{010\cdot 1} \\ 2 - 2p_{000\cdot 1} - p_{100\cdot 0} - p_{100\cdot 1} - p_{010\cdot 1} - p_{001\cdot 1} - p_{101\cdot 1} \end{array} \right\}.$$

Result 1d:

The bounds given below are valid and tight for $\Delta E\{Y(1 \to 0, M_1(1), 1)\} = E\{Y(1, M_1(1), 1)\} - E\{Y(0, M_1(1), 1)\}$ under Figure 1a.

$$\Delta E\{Y(1 \to 0, M_1(1), 1)\} \geq$$
$$\max \left\{ \begin{array}{l} -2p_{000\cdot 1} - 2p_{100\cdot 1} - p_{010\cdot 1} - p_{110\cdot 1} - 2p_{001\cdot 1} - p_{101\cdot 1} + p_{011\cdot 0} - p_{011\cdot 1} \\ -1 - p_{010\cdot 1} - p_{110\cdot 1} + p_{001\cdot 0} + p_{101\cdot 1} - p_{011\cdot 1} \\ -p_{000\cdot 1} - p_{100\cdot 1} - p_{010\cdot 1} - p_{110\cdot 1} - p_{001\cdot 1} - p_{011\cdot 1} \end{array} \right\},$$

and

$$\Delta E\{Y(1 \rightarrow 0, M_1(1), 1)\} \leq$$
$$\min \left\{ \begin{array}{l} p_{000\cdot 0} + p_{000\cdot 1} + p_{100\cdot 0} + p_{100\cdot 1} + p_{010\cdot 0} + p_{110\cdot 0} + p_{001\cdot 0} + p_{101\cdot 0} + p_{101\cdot 1} + p_{011\cdot 0} - p_{011\cdot 1} \\ 1 - p_{001\cdot 1} - p_{011\cdot 1} \\ 2 - p_{000\cdot 1} - p_{100\cdot 1} - 2p_{001\cdot 1} - p_{101\cdot 0} - p_{101\cdot 1} - p_{011\cdot 1} \end{array} \right\}.$$

Result 2b:
The bounds given below are valid and tight for $\Delta E\{Y(1 \rightarrow 0, 0, M_2(0,1))\} = E\{Y(1,0,M_2(0,1))\} - E\{Y(0,0,M_2(0,1))\}$ under Figure 1b.

$$\Delta E\{Y(1 \rightarrow 0, 0, M_2(0,1))\} \geq$$
$$\max \left\{ \begin{array}{l} -1 - p_{100\cdot 0} - p_{010\cdot 0} - p_{110\cdot 0} - p_{001\cdot 0} - p_{101\cdot 0} + p_{101\cdot 1} \\ -2 + p_{100\cdot 1} + p_{010\cdot 0} + p_{110\cdot 0} + p_{001\cdot 0} \\ -1 \end{array} \right\},$$

and

$$\Delta E\{Y(1 \rightarrow 0, 0, M_2(0,1))\} \leq$$
$$\min \left\{ \begin{array}{l} 2 - p_{000\cdot 1} - p_{010\cdot 0} - p_{110\cdot 0} - p_{101\cdot 0} \\ 1 + p_{000\cdot 0} + p_{010\cdot 0} + p_{110\cdot 0} + p_{001\cdot 0} - p_{001\cdot 1} + p_{101\cdot 0} \\ 1 \end{array} \right\}.$$

Result 2c:
The bounds given below are valid and tight for $\Delta E\{Y(1 \rightarrow 0, 0, M_2(1,0))\} = E\{Y(1,0,M_2(1,0))\} - E\{Y(0,0,M_2(1,0))\}$ under Figure 1b.

$$\Delta E\{Y(1 \rightarrow 0, 0, M_2(1,0))\} \geq$$
$$\max \left\{ \begin{array}{l} -2 + p_{100\cdot 1} + p_{001\cdot 0} + p_{001\cdot 1} + 2p_{101\cdot 1} \\ -2 + p_{000\cdot 0} + p_{000\cdot 1} + 2p_{100\cdot 1} + p_{101\cdot 1} \\ -1 + p_{100\cdot 1} + p_{101\cdot 1} \end{array} \right\},$$

and

$$\Delta E\{Y(1 \rightarrow 0, 0, M_2(1,0))\} \leq$$
$$\min \left\{ \begin{array}{l} 2 - p_{000\cdot 1} - 2p_{001\cdot 1} - p_{101\cdot 0} - p_{101\cdot 1} \\ 1 - p_{000\cdot 1} - p_{001\cdot 1} \\ 2 - 2p_{000\cdot 1} - p_{100\cdot 0} - p_{100\cdot 1} - p_{001\cdot 1} \end{array} \right\}.$$

Result 2d:
The bounds given below are valid and tight for $\Delta E\{Y(1 \rightarrow 0, 0, M_2(1,1))\} = E\{Y(1,0,M_2(1,1))\} - E\{Y(0,0,M_2(1,1))\}$ under Figure 1b.

$$\Delta E\{Y(1 \rightarrow 0, 0, M_2(1,1))\} \geq$$
$$\max \left\{ \begin{array}{l} -1 - p_{000\cdot 1} - p_{010\cdot 1} - p_{110\cdot 1} + p_{001\cdot 0} - p_{001\cdot 1} - p_{101\cdot 1} \\ -2 + p_{000\cdot 0} + p_{010\cdot 1} + p_{110\cdot 1} + p_{101\cdot 1} \\ -1 \end{array} \right\},$$

and

$$\Delta E\{Y(1 \rightarrow 0, 0, M_2(1,1))\} \leq$$
$$\min \left\{ \begin{array}{l} 1 + p_{100\cdot 1} + p_{010\cdot 1} + p_{110\cdot 1} + p_{001\cdot 1} - p_{101\cdot 0} + p_{101\cdot 1} \\ 2 - p_{100\cdot 0} - p_{010\cdot 1} - p_{110\cdot 1} - p_{001\cdot 1} \\ 1 \end{array} \right\}.$$

Result 2e:
The bounds given below are valid and tight for $\Delta E\{Y(1 \rightarrow 0, 1, M_2(0,0))\} = E\{Y(1, 1, M_2(0,0))\} - E\{Y(0, 1, M_2(0,0))\}$ under Figure 1b.

$$\Delta E\{Y(1 \rightarrow 0, 1, M_2(0,0))\} \geq$$
$$\max \left\{ \begin{array}{l} -1 - p_{000\cdot 1} - p_{100\cdot 1} + p_{010\cdot 0} - p_{010\cdot 1} - p_{110\cdot 1} + p_{001\cdot 0} - p_{001\cdot 1} + p_{101\cdot 0} - p_{101\cdot 1} - p_{011\cdot 1} \\ -2 + p_{000\cdot 0} + p_{100\cdot 0} + p_{110\cdot 1} + p_{011\cdot 0} \\ -1 \end{array} \right\},$$

and

$$\Delta E\{Y(1 \rightarrow 0, 1, M_2(0,0))\} \leq$$
$$\min \left\{ \begin{array}{l} 1 + p_{010\cdot 0} - p_{010\cdot 1} + p_{110\cdot 0} + p_{001\cdot 0} + p_{101\cdot 0} + p_{011\cdot 0} \\ 2 - p_{110\cdot 0} - p_{001\cdot 0} - p_{101\cdot 0} - p_{011\cdot 1} \\ 1 \end{array} \right\}.$$

Result 2f:
The bounds given below are valid and tight for $\Delta E\{Y(1 \rightarrow 0, 1, M_2(0,1))\} = E\{Y(1, 1, M_2(0,1))\} - E\{Y(0, 1, M_2(0,1))\}$ under Figure 1b.

$$\Delta E\{Y(1 \rightarrow 0, 1, M_2(0,1))\} \geq$$
$$\max \left\{ \begin{array}{l} -p_{000\cdot 0} - p_{100\cdot 0} - p_{100\cdot 1} - p_{110\cdot 0} - p_{001\cdot 0} - p_{101\cdot 0} + p_{011\cdot 0} - 1 + p_{111\cdot 1} \\ -2 + 2p_{010\cdot 0} + p_{110\cdot 0} + p_{110\cdot 1} + p_{011\cdot 0} \\ -1 + p_{010\cdot 0} + p_{011\cdot 0} \end{array} \right\},$$

and

$$\Delta E\{Y(1 \rightarrow 0, 1, M_2(0,1))\} \leq$$
$$\min \left\{ \begin{array}{l} 2p_{000\cdot 0} + 2p_{100\cdot 0} + 2p_{010\cdot 0} + p_{110\cdot 0} + 2p_{001\cdot 0} + 2p_{101\cdot 0} + p_{011\cdot 0} - p_{011\cdot 1} \\ p_{000\cdot 0} + p_{100\cdot 0} + p_{010\cdot 0} + p_{001\cdot 0} + p_{101\cdot 0} + p_{011\cdot 0} \\ 1 + p_{000\cdot 0} + p_{100\cdot 0} - p_{010\cdot 1} - p_{110\cdot 0} + p_{001\cdot 0} + p_{101\cdot 0} + p_{011\cdot 0} \end{array} \right\}.$$

Result 2g:
The bounds given below are valid and tight for $\Delta E\{Y(1 \to 0, 1, M_2(1,0))\} = E\{Y(1,1,M_2(1,0))\} - E\{Y(0,1,M_2(1,0))\}$ under Figure 1b.

$$\Delta E\{Y(1 \to 0, 1, M_2(1,0))\} \geq \max \left\{ \begin{array}{l} -2 + p_{110\cdot1} + p_{001\cdot1} + p_{101\cdot1} + p_{011\cdot0} \\ -1 + p_{010\cdot0} - p_{010\cdot1} - p_{110\cdot1} - p_{001\cdot1} - p_{101\cdot1} - p_{011\cdot1} \\ -1 \end{array} \right\},$$

and

$$\Delta E\{Y(1 \to 0, 1, M_2(1,0))\} \leq \min \left\{ \begin{array}{l} 2 - p_{000\cdot1} - p_{100\cdot1} - p_{110\cdot0} - p_{011\cdot1} \\ 1 + p_{000\cdot0} + p_{100\cdot0} + p_{010\cdot0} - p_{010\cdot1} + p_{110\cdot0} + p_{001\cdot0} - p_{001\cdot1} + p_{101\cdot0} - p_{101\cdot1} + p_{011\cdot0} \\ 1 \end{array} \right\}.$$

Result 2h:
The bounds given below are valid and tight for $\Delta E\{Y(1 \to 0, 1, M_2(1,1))\} = E\{Y(1,1,M_2(1,1))\} - E\{Y(0,1,M_2(1,1))\}$ under Figure 1b.

$$\Delta E\{Y(1 \to 0, 1, M_2(1,1))\} \geq \max \left\{ \begin{array}{l} -2p_{000\cdot1} - 2p_{100\cdot1} - 2p_{010\cdot1} - p_{110\cdot1} - 2p_{001\cdot1} - 2p_{101\cdot1} + p_{011\cdot0} - p_{011\cdot1} \\ -1 - p_{000\cdot1} - p_{100\cdot1} + p_{010\cdot0} + p_{110\cdot1} - p_{001\cdot1} - p_{101\cdot1} - p_{011\cdot1} \\ -p_{000\cdot1} - p_{100\cdot1} - p_{010\cdot1} - p_{001\cdot1} - p_{101\cdot1} - p_{011\cdot1} \end{array} \right\},$$

and

$$\Delta E\{Y(1 \to 0, 1, M_2(1,1))\} \leq \min \left\{ \begin{array}{l} p_{000\cdot1} + p_{100\cdot1} + p_{110\cdot1} + p_{001\cdot1} + p_{101\cdot1} + 1 - p_{111\cdot0} - p_{011\cdot1} \\ 1 - p_{010\cdot1} - p_{011\cdot1} \\ 2 - 2p_{010\cdot1} - p_{110\cdot0} - p_{110\cdot1} - p_{011\cdot1} \end{array} \right\}.$$

Result 3b:
The bounds given below are valid and tight for $\Delta E\{Y(1 \to 0, M_1(0), M_2(0,1))\} = E\{Y(1, M_1(0), M_2(0,1))\} - E\{Y(0, M_1(0), M_2(0,1))\}$ under Figure 1b.

$$\Delta E\{Y(1 \to 0, M_1(0), M_2(0,1))\} \geq$$
$$\max \left\{ \begin{array}{l} -p_{000\cdot 0} - p_{100\cdot 0} - p_{110\cdot 0} - p_{001\cdot 0} - p_{101\cdot 0} + p_{011\cdot 0} - 1 + p_{111\cdot 1} \\ -2 + 2p_{010\cdot 0} + p_{110\cdot 0} + p_{110\cdot 1} + p_{011\cdot 0} \\ -1 + p_{010\cdot 0} + p_{011\cdot 0} \\ -2 + p_{000\cdot 0} + p_{010\cdot 0} + p_{101\cdot 1} + p_{011\cdot 0} \\ -2 + p_{100\cdot 1} + p_{010\cdot 0} + p_{001\cdot 0} + p_{011\cdot 0} \end{array} \right\},$$

and

$$\Delta E\{Y(1 \to 0, M_1(0), M_2(0,1))\} \leq$$
$$\min \left\{ \begin{array}{l} 2p_{000\cdot 0} + 2p_{100\cdot 0} + 2p_{010\cdot 0} + p_{110\cdot 0} + 2p_{001\cdot 0} + 2p_{101\cdot 0} + p_{011\cdot 0} - p_{011\cdot 1} \\ 1 + p_{000\cdot 0} + p_{010\cdot 0} + p_{001\cdot 0} - p_{001\cdot 1} + p_{101\cdot 0} + p_{011\cdot 0} \\ 1 + p_{000\cdot 0} - p_{000\cdot 1} + p_{100\cdot 0} + p_{010\cdot 0} + p_{001\cdot 0} + p_{011\cdot 0} \\ p_{000\cdot 0} + p_{100\cdot 0} + p_{010\cdot 0} + p_{001\cdot 0} + p_{101\cdot 0} + p_{011\cdot 0} \\ 1 + p_{000\cdot 0} + p_{100\cdot 0} - p_{010\cdot 1} - p_{110\cdot 0} + p_{001\cdot 0} + p_{101\cdot 0} + p_{011\cdot 0} \end{array} \right\}.$$

Result 3c:
The bounds given below are valid and tight for $\Delta E\{Y(1 \to, M_1(0), M_2(1,0))\} = E\{Y(1, M_1(0), M_2(1,0))\} - E\{Y(0, M_1(0), M_2(1,0))\}$ under Figure 1b.

$$\Delta E\{Y(1 \to, M_1(0), M_2(1,0))\} \geq$$
$$\max \left\{ \begin{array}{l} -3 + p_{000\cdot 0} + p_{100\cdot 0} + p_{100\cdot 1} + 2p_{001\cdot 0} + p_{001\cdot 1} + p_{101\cdot 0} + 2p_{101\cdot 1} + p_{011\cdot 0} \\ -3 + 2p_{000\cdot 0} + p_{000\cdot 1} + p_{100\cdot 0} + 2p_{100\cdot 1} + p_{010\cdot 0} + p_{001\cdot 0} + p_{101\cdot 0} + p_{101\cdot 1} \\ -2 + p_{000\cdot 0} + p_{100\cdot 0} + p_{001\cdot 0} + p_{101\cdot 0} + p_{101\cdot 1} + p_{011\cdot 0} \\ -2 + p_{001\cdot 0} + p_{001\cdot 1} + p_{101\cdot 1} + p_{011\cdot 0} \\ -2 + p_{110\cdot 1} + p_{001\cdot 1} + p_{101\cdot 1} + p_{011\cdot 0} \\ -2 + p_{000\cdot 0} + p_{100\cdot 0} + p_{100\cdot 1} + p_{001\cdot 0} + p_{101\cdot 0} + p_{101\cdot 1} \\ -2 + p_{100\cdot 1} + p_{001\cdot 0} + p_{001\cdot 1} + p_{101\cdot 1} \\ -1 + p_{010\cdot 0} - p_{010\cdot 1} - p_{110\cdot 1} - p_{001\cdot 1} - p_{101\cdot 1} - p_{011\cdot 1} \\ -2 + p_{000\cdot 0} + p_{100\cdot 0} + p_{100\cdot 1} + p_{010\cdot 0} + p_{001\cdot 0} + p_{101\cdot 0} \\ -2 + p_{000\cdot 0} + p_{000\cdot 1} + p_{100\cdot 1} + p_{010\cdot 0} \\ -2 + p_{000\cdot 0} + p_{000\cdot 1} + p_{100\cdot 1} + p_{101\cdot 1} \\ -1 \end{array} \right\},$$

and

$$\Delta E\{Y(1 \rightarrow, M_1(0), M_2(1,0))\} \leq$$

$$\min \left\{ \begin{array}{l}
2 - p_{000\cdot 1} + p_{010\cdot 0} + p_{110\cdot 0} - 2p_{001\cdot 1} - p_{101\cdot 0} - p_{101\cdot 1} + p_{011\cdot 0} \\
1 \\
1 + p_{000\cdot 0} + p_{100\cdot 0} + p_{010\cdot 0} + p_{110\cdot 0} + p_{001\cdot 0} - p_{001\cdot 1} - p_{101\cdot 1} + p_{011\cdot 0} \\
1 + p_{000\cdot 0} + p_{100\cdot 0} + p_{010\cdot 0} - p_{010\cdot 1} + p_{110\cdot 0} + p_{001\cdot 0} - p_{001\cdot 1} + p_{101\cdot 0} - p_{101\cdot 1} + p_{011\cdot 0} \\
2 - p_{000\cdot 1} - p_{100\cdot 0} - p_{100\cdot 1} - p_{001\cdot 1} \\
2 - p_{000\cdot 1} - p_{001\cdot 1} - p_{101\cdot 0} - p_{101\cdot 1} \\
2 - p_{000\cdot 1} - p_{100\cdot 1} - p_{110\cdot 0} - p_{011\cdot 1} \\
2 - p_{000\cdot 1} - p_{100\cdot 0} - p_{100\cdot 1} - p_{110\cdot 0} \\
1 + p_{010\cdot 0} + p_{110\cdot 0} - p_{001\cdot 1} + p_{011\cdot 0} \\
2 - p_{000\cdot 0} - p_{000\cdot 1} - p_{100\cdot 0} - p_{110\cdot 0} - p_{001\cdot 0} - p_{101\cdot 0} \\
2 - p_{000\cdot 0} - p_{000\cdot 1} - p_{100\cdot 0} - p_{001\cdot 0} - p_{001\cdot 1} - p_{101\cdot 0} \\
3 - p_{000\cdot 0} - 2p_{000\cdot 1} - 2p_{100\cdot 0} - p_{100\cdot 1} - p_{110\cdot 0} - p_{001\cdot 0} - p_{001\cdot 1} - p_{101\cdot 0}
\end{array} \right\}.$$

Result 3d:
The bounds given below are valid and tight for $\Delta E\{Y(1 \rightarrow, M_1(0), M_2(1,1))\} = E\{Y(1, M_1(0), M_2(1,1))\} - E\{Y(0, M_1(0), M_2(1,1))\}$ under Figure 1b.

$$\Delta E\{Y(1 \rightarrow, M_1(0), M_2(1,1))\} \geq$$

$$\max \left\{ \begin{array}{l}
-p_{000\cdot 0} - 2p_{000\cdot 1} - p_{100\cdot 0} - 2p_{100\cdot 1} - 2p_{010\cdot 1} - p_{110\cdot 1} - 2p_{001\cdot 1} - p_{101\cdot 0} - 2p_{101\cdot 1} + p_{011\cdot 0} - p_{011\cdot 1} \\
-1 - p_{000\cdot 1} - p_{100\cdot 0} - p_{100\cdot 1} + p_{010\cdot 0} + p_{110\cdot 1} - p_{001\cdot 0} - p_{001\cdot 1} - p_{101\cdot 0} - p_{101\cdot 1} - p_{011\cdot 1} \\
-p_{000\cdot 0} - p_{000\cdot 1} - p_{100\cdot 0} - p_{100\cdot 1} - p_{010\cdot 1} - p_{110\cdot 1} - p_{001\cdot 1} - p_{101\cdot 0} - p_{101\cdot 1} - p_{011\cdot 1} \\
-p_{000\cdot 0} - p_{000\cdot 1} - p_{100\cdot 0} - p_{100\cdot 1} - p_{010\cdot 1} - p_{001\cdot 0} - p_{001\cdot 1} - p_{101\cdot 0} - p_{101\cdot 1} - p_{011\cdot 1} \\
-1 - p_{000\cdot 1} - p_{100\cdot 1} - p_{010\cdot 1} - p_{110\cdot 1} + p_{001\cdot 0} - p_{001\cdot 1} - p_{101\cdot 1} + p_{011\cdot 0} \\
-1 - p_{000\cdot 1} - p_{100\cdot 1} - p_{010\cdot 1} - p_{001\cdot 1} - p_{101\cdot 1} + p_{011\cdot 0} \\
-1 - p_{000\cdot 1} - p_{010\cdot 1} - p_{110\cdot 1} + p_{001\cdot 0} - p_{001\cdot 1} - p_{101\cdot 1} \\
-1 - p_{100\cdot 0} + p_{110\cdot 1} - p_{001\cdot 0} - p_{101\cdot 0} \\
-1 - p_{000\cdot 1} - p_{100\cdot 1} + p_{010\cdot 0} - p_{001\cdot 1} - p_{101\cdot 1} - p_{011\cdot 1} \\
-2 + p_{000\cdot 0} + p_{010\cdot 0} + p_{010\cdot 1} + p_{110\cdot 1} \\
-2 + p_{000\cdot 0} + p_{010\cdot 1} + p_{110\cdot 1} + p_{101\cdot 1} \\
-1
\end{array} \right\},$$

and

$$\Delta\mathrm{E}\{Y(1 \rightarrow, M_1(0), M_2(1,1))\} \leq$$

$$\min \left\{ \begin{array}{l} p_{000\cdot 0} + p_{000\cdot 1} + p_{100\cdot 0} + p_{100\cdot 1} + p_{110\cdot 1} + p_{001\cdot 1} + p_{101\cdot 1} + 1 - p_{111\cdot 0} - p_{011\cdot 1} \\ 1 \\ p_{000\cdot 0} + p_{000\cdot 1} + p_{100\cdot 0} + p_{100\cdot 1} + p_{010\cdot 0} + p_{010\cdot 1} + p_{110\cdot 0} + p_{110\cdot 1} + p_{001\cdot 0} + p_{001\cdot 1} + p_{101\cdot 1} + p_{011\cdot 0} \\ 2 - p_{010\cdot 1} - p_{110\cdot 0} - p_{110\cdot 1} - p_{011\cdot 1} \\ p_{000\cdot 0} + p_{000\cdot 1} + p_{100\cdot 0} + p_{100\cdot 1} + p_{010\cdot 0} + p_{110\cdot 0} + p_{110\cdot 1} + p_{001\cdot 0} + p_{001\cdot 1} + p_{101\cdot 0} + p_{101\cdot 1} + p_{011\cdot 0} \\ 1 + p_{000\cdot 0} + p_{100\cdot 0} + p_{001\cdot 0} - p_{011\cdot 1} \\ 1 + p_{000\cdot 0} + p_{100\cdot 0} - p_{010\cdot 1} + p_{001\cdot 0} + p_{101\cdot 0} - p_{011\cdot 1} \\ 1 + p_{000\cdot 0} - p_{010\cdot 1} + p_{001\cdot 0} + p_{101\cdot 0} \\ 2 - p_{100\cdot 0} - p_{010\cdot 1} - p_{110\cdot 0} - p_{110\cdot 1} \\ 2 - p_{100\cdot 0} - p_{010\cdot 1} - p_{110\cdot 1} - p_{001\cdot 1} \\ 1 + p_{100\cdot 1} + p_{010\cdot 1} + p_{110\cdot 1} + p_{001\cdot 1} - p_{101\cdot 0} + p_{101\cdot 1} \\ 2 + p_{000\cdot 0} - 2p_{010\cdot 1} - p_{110\cdot 0} - p_{110\cdot 1} + p_{001\cdot 0} + p_{101\cdot 0} - p_{011\cdot 1} \end{array} \right\}.$$

Result 3e:
The bounds given below are valid and tight for $\Delta\mathrm{E}\{Y(1 \rightarrow 0, M_1(1), M_2(0,0))\} = E\{Y(1, M_1(1), M_2(0,0))\} -$ $E\{Y(0, M_1(1), M_2(0,0))\}$ under Figure 1b.

$$\Delta\mathrm{E}\{Y(1 \rightarrow 0, M_1(1), M_2(0,0))\} \geq$$

$$\max \left\{ \begin{array}{l} -2 + p_{000\cdot 0} - p_{010\cdot 1} - p_{110\cdot 1} + 2p_{001\cdot 0} + p_{101\cdot 0} + p_{101\cdot 1} - p_{011\cdot 1} \\ -3 + 2p_{000\cdot 0} + p_{000\cdot 1} + p_{100\cdot 0} + 2p_{100\cdot 1} + p_{110\cdot 1} + p_{001\cdot 0} + p_{001\cdot 1} + p_{101\cdot 1} \\ -1 - p_{000\cdot 1} - p_{100\cdot 1} - p_{010\cdot 1} - p_{110\cdot 1} + p_{001\cdot 0} - p_{001\cdot 1} + p_{101\cdot 0} - p_{011\cdot 1} \\ -1 - p_{000\cdot 1} - p_{100\cdot 1} + p_{010\cdot 0} - p_{010\cdot 1} - p_{110\cdot 1} + p_{001\cdot 0} - p_{001\cdot 1} + p_{101\cdot 0} - p_{101\cdot 1} - p_{011\cdot 1} \\ -2 + p_{000\cdot 0} + p_{001\cdot 0} + p_{101\cdot 0} + p_{101\cdot 1} \\ -1 - p_{010\cdot 1} - p_{110\cdot 1} + p_{001\cdot 0} - p_{011\cdot 1} \\ -2 + p_{000\cdot 0} + p_{000\cdot 1} + p_{100\cdot 1} + p_{001\cdot 0} + p_{001\cdot 1} + p_{101\cdot 1} \\ -2 + p_{000\cdot 0} + p_{100\cdot 0} + p_{110\cdot 1} + p_{011\cdot 0} \\ -2 + p_{000\cdot 0} + p_{100\cdot 0} + p_{100\cdot 1} + p_{001\cdot 0} \\ -2 + p_{000\cdot 0} + p_{100\cdot 0} + p_{100\cdot 1} + p_{110\cdot 1} \\ -2 + p_{000\cdot 0} + p_{000\cdot 1} + p_{100\cdot 1} + p_{110\cdot 1} + p_{001\cdot 1} + p_{101\cdot 1} \\ -1 \end{array} \right\},$$

and

$$\Delta E\{Y(1 \to 0, M_1(1), M_2(0,0))\} \leq$$

$$\min \left\{ \begin{array}{l} 3 - p_{000\cdot 1} - p_{100\cdot 0} - p_{100\cdot 1} - p_{001\cdot 0} - 2p_{001\cdot 1} - 2p_{101\cdot 0} - p_{101\cdot 1} - p_{011\cdot 1} \\ 1 \\ 2 - p_{001\cdot 0} - p_{001\cdot 1} - p_{101\cdot 0} - p_{011\cdot 1} \\ 2 - p_{110\cdot 0} - p_{001\cdot 0} - p_{101\cdot 0} - p_{011\cdot 1} \\ 2 - p_{100\cdot 0} - p_{001\cdot 0} - p_{001\cdot 1} - p_{101\cdot 0} \\ 2 - p_{000\cdot 1} - p_{100\cdot 0} - p_{100\cdot 1} - p_{010\cdot 1} - p_{001\cdot 1} - p_{101\cdot 1} \\ 2 - p_{000\cdot 1} - p_{100\cdot 1} - p_{001\cdot 1} - p_{101\cdot 0} - p_{101\cdot 1} - p_{011\cdot 1} \\ 2 - p_{000\cdot 1} - p_{100\cdot 0} - p_{100\cdot 1} - p_{001\cdot 1} - p_{101\cdot 0} - p_{101\cdot 1} \\ 1 + p_{010\cdot 0} - p_{010\cdot 1} + p_{110\cdot 0} + p_{001\cdot 0} + p_{101\cdot 0} + p_{011\cdot 0} \\ 2 - p_{000\cdot 0} - p_{000\cdot 1} - p_{100\cdot 0} - p_{101\cdot 0} \\ 2 - p_{000\cdot 0} - p_{000\cdot 1} - p_{100\cdot 0} - p_{010\cdot 1} \\ 3 - p_{000\cdot 0} - 2p_{000\cdot 1} - 2p_{100\cdot 0} - p_{100\cdot 1} - p_{010\cdot 1} - p_{001\cdot 1} - p_{101\cdot 0} - p_{101\cdot 1} \end{array} \right\}.$$

Result 3f:
The bounds given below are valid and tight for $\Delta E\{Y(1 \to 0, M_1(1), M_2(0,1))\} = E\{Y(1, M_1(1), M_2(0,1))\} -$ $E\{Y(0, M_1(1), M_2(0,1))\}$ under Figure 1b.

$$\Delta E\{Y(1 \to 0, M_1(1), M_2(0,1))\} \geq$$

$$\max \left\{ \begin{array}{l} -p_{000\cdot 0} - p_{000\cdot 1} - p_{100\cdot 0} - p_{100\cdot 1} - p_{110\cdot 0} - p_{001\cdot 0} - p_{001\cdot 1} - p_{101\cdot 0} + p_{011\cdot 0} - 1 + p_{111\cdot 1} \\ -2 - p_{000\cdot 1} + 2p_{010\cdot 0} + p_{110\cdot 0} + p_{110\cdot 1} - p_{001\cdot 1} - p_{101\cdot 1} + p_{011\cdot 0} \\ -p_{000\cdot 0} - p_{000\cdot 1} - p_{100\cdot 0} - p_{100\cdot 1} - p_{010\cdot 0} - p_{010\cdot 1} - p_{110\cdot 0} - p_{110\cdot 1} - p_{001\cdot 0} - p_{001\cdot 1} - p_{101\cdot 0} - p_{011\cdot 1} \\ -p_{000\cdot 0} - p_{000\cdot 1} - p_{100\cdot 0} - p_{100\cdot 1} - p_{010\cdot 1} - p_{110\cdot 0} - p_{110\cdot 1} - p_{001\cdot 0} - p_{001\cdot 1} - p_{101\cdot 0} - p_{101\cdot 1} - p_{011\cdot 1} \\ -1 - p_{000\cdot 1} - p_{100\cdot 1} - p_{001\cdot 1} + p_{011\cdot 0} \\ -1 - p_{000\cdot 1} - p_{100\cdot 1} + p_{010\cdot 0} - p_{001\cdot 1} - p_{101\cdot 1} + p_{011\cdot 0} \\ -1 - p_{100\cdot 0} - p_{010\cdot 0} - p_{110\cdot 0} - p_{001\cdot 0} - p_{101\cdot 0} + p_{101\cdot 1} \\ -2 + p_{010\cdot 0} + p_{110\cdot 0} + p_{110\cdot 1} + p_{011\cdot 0} \\ -2 + p_{100\cdot 1} + p_{010\cdot 0} + p_{110\cdot 0} + p_{110\cdot 1} \\ -1 - p_{000\cdot 1} + p_{010\cdot 0} - p_{001\cdot 1} - p_{101\cdot 1} \\ -2 + p_{100\cdot 1} + p_{010\cdot 0} + p_{110\cdot 0} + p_{001\cdot 0} \\ -1 \end{array} \right\},$$

and

$$\Delta E\{Y(1 \to 0, M_1(1), M_2(0,1))\} \leq$$

$$\min \left\{ \begin{array}{l} p_{000\cdot 0} + p_{000\cdot 1} + p_{100\cdot 0} + p_{100\cdot 1} + p_{010\cdot 0} + p_{001\cdot 0} + p_{101\cdot 0} + p_{101\cdot 1} + 1 - p_{111\cdot 0} - p_{011\cdot 1} \\ 1 \\ 1 + p_{000\cdot 0} + p_{100\cdot 0} + p_{010\cdot 0} + p_{110\cdot 0} + p_{001\cdot 0} - p_{001\cdot 1} + p_{101\cdot 0} - p_{011\cdot 1} \\ 1 + p_{000\cdot 0} + p_{100\cdot 0} + p_{010\cdot 0} + p_{001\cdot 0} + p_{101\cdot 0} - p_{011\cdot 1} \\ p_{000\cdot 0} + p_{000\cdot 1} + p_{100\cdot 0} + p_{100\cdot 1} + p_{010\cdot 0} + p_{110\cdot 0} + p_{001\cdot 0} + p_{101\cdot 0} + p_{101\cdot 1} + p_{011\cdot 0} \\ p_{000\cdot 0} + p_{000\cdot 1} + p_{100\cdot 0} + p_{100\cdot 1} + p_{010\cdot 0} + p_{001\cdot 0} + p_{001\cdot 1} + p_{101\cdot 0} + p_{101\cdot 1} + p_{011\cdot 0} \\ 1 + p_{000\cdot 0} + p_{100\cdot 0} - p_{010\cdot 1} + p_{001\cdot 0} + p_{101\cdot 0} + p_{011\cdot 0} \\ 1 + p_{000\cdot 0} + p_{010\cdot 0} + p_{110\cdot 0} + p_{001\cdot 0} - p_{001\cdot 1} + p_{101\cdot 0} \\ 1 + p_{100\cdot 1} - p_{110\cdot 0} + p_{001\cdot 1} + p_{101\cdot 1} \\ 2 - p_{000\cdot 1} - p_{010\cdot 0} - p_{010\cdot 1} - p_{110\cdot 0} \\ 2 - p_{000\cdot 1} - p_{010\cdot 0} - p_{110\cdot 0} - p_{101\cdot 0} \\ 1 + p_{000\cdot 0} + p_{100\cdot 0} + p_{100\cdot 1} - p_{010\cdot 1} - p_{110\cdot 0} + p_{001\cdot 0} + p_{001\cdot 1} + p_{101\cdot 0} + p_{101\cdot 1} + p_{011\cdot 0} \end{array} \right\}.$$

Result 3g:
The bounds given below are valid and tight for $\Delta E\{Y(1, M_1(1), M_2(1,0))\} = E\{Y(1, M_1(1), M_2(1,0))\} - E\{Y(0, M_1(1), M_2(1,0))\}$ under Figure 1b.

$$\Delta E\{Y(1, M_1(1), M_2(1,0))\} \geq$$

$$\max \left\{ \begin{array}{l} -2 + p_{100\cdot 1} + p_{001\cdot 0} + p_{001\cdot 1} + 2p_{101\cdot 1} \\ -2 + p_{000\cdot 0} + p_{000\cdot 1} + 2p_{100\cdot 1} + p_{101\cdot 1} \\ -1 + p_{100\cdot 1} + p_{101\cdot 1} \\ -2 + p_{100\cdot 1} + p_{110\cdot 1} + p_{101\cdot 1} + p_{011\cdot 0} \\ -1 - p_{000\cdot 1} + p_{010\cdot 0} - p_{010\cdot 1} - p_{110\cdot 1} - p_{001\cdot 1} - p_{011\cdot 1} \end{array} \right\},$$

and

$$\Delta E\{Y(1, M_1(1), M_2(1,0))\} \leq$$

$$\min \left\{ \begin{array}{l} 2 - p_{000\cdot 1} - 2p_{001\cdot 1} - p_{101\cdot 0} - p_{101\cdot 1} \\ 1 + p_{000\cdot 0} - p_{000\cdot 1} + p_{100\cdot 0} + p_{010\cdot 0} - p_{010\cdot 1} + p_{110\cdot 0} + p_{001\cdot 0} - p_{001\cdot 1} + p_{101\cdot 0} + p_{011\cdot 0} \\ 2 - p_{000\cdot 1} - p_{110\cdot 0} - p_{001\cdot 1} - p_{011\cdot 1} \\ 1 - p_{000\cdot 1} - p_{001\cdot 1} \\ 2 - 2p_{000\cdot 1} - p_{100\cdot 0} - p_{100\cdot 1} - p_{001\cdot 1} \end{array} \right\}.$$

Result 3h:
The bounds given below are valid and tight for $\Delta E\{Y(1 \to 0, M_1(1), M_2(1,1))\} = E\{Y(1, M_1(1), M_2(1,1))\} - E\{Y(0, M_1(1), M_2(1,1))\}$ under Figure 1b.

$$
\Delta\mathrm{E}\{Y(1\to 0, M_1(1), M_2(1,1))\} \geq
\max\left\{\begin{array}{l}
-2p_{000\cdot 1}-2p_{100\cdot 1}-2p_{010\cdot 1}-p_{110\cdot 1}-2p_{001\cdot 1}-2p_{101\cdot 1}+p_{011\cdot 0}-p_{011\cdot 1}\\
-1-p_{000\cdot 1}-p_{100\cdot 1}+p_{010\cdot 0}+p_{110\cdot 1}-p_{001\cdot 1}-p_{101\cdot 1}-p_{011\cdot 1}\\
-p_{000\cdot 1}-p_{100\cdot 1}-p_{010\cdot 1}-p_{001\cdot 1}-p_{101\cdot 1}-p_{011\cdot 1}\\
-1-p_{000\cdot 1}-p_{010\cdot 1}+p_{001\cdot 0}-p_{001\cdot 1}-p_{101\cdot 1}-p_{011\cdot 1}\\
-1+p_{000\cdot 0}-p_{000\cdot 1}-p_{100\cdot 1}-p_{010\cdot 1}-p_{001\cdot 1}-p_{011\cdot 1}
\end{array}\right\},
$$

and

$$
\Delta\mathrm{E}\{Y(1\to 0, M_1(1), M_2(1,1))\} \leq
\min\left\{\begin{array}{l}
p_{000\cdot 1}+p_{100\cdot 1}+p_{110\cdot 1}+p_{001\cdot 1}+p_{101\cdot 1}+1-p_{111\cdot 0}-p_{011\cdot 1}\\
2-p_{100\cdot 0}-p_{010\cdot 1}-p_{001\cdot 1}-p_{011\cdot 1}\\
2-p_{000\cdot 1}-p_{010\cdot 1}-p_{101\cdot 0}-p_{011\cdot 1}\\
1-p_{010\cdot 1}-p_{011\cdot 1}\\
2-2p_{010\cdot 1}-p_{110\cdot 0}-p_{110\cdot 1}-p_{011\cdot 1}
\end{array}\right\}.
$$

Result 4b:
The bounds given below are valid and tight for $\Delta E\{Y(1 \to 0, 0, M_2(0, M_1(1)))\} = E\{Y(1, 0, M_2(0, M_1(1)))\}- E\{Y(0, 0, M_2(0, M_1(1)))\}$ under Figure 1b.

$$\Delta E\{Y(1 \to 0, 0, M_2(0, M_1(1)))\} \geq \max\left\{\begin{array}{l} -3 + p_{000\cdot0} + p_{000\cdot1} + p_{100\cdot1} + 2p_{001\cdot0} + p_{001\cdot1} + p_{101\cdot0} + 2p_{101\cdot1} \\ -3 + 2p_{000\cdot0} + p_{000\cdot1} + p_{100\cdot0} + 2p_{100\cdot1} + p_{001\cdot0} + p_{001\cdot1} + p_{101\cdot1} \\ -2 + p_{000\cdot0} + p_{001\cdot0} + p_{101\cdot0} + p_{101\cdot1} \\ -2 + p_{000\cdot0} + p_{000\cdot1} + p_{100\cdot1} + p_{001\cdot0} + p_{001\cdot1} + p_{101\cdot1} \\ -2 + p_{000\cdot0} + p_{100\cdot0} + p_{100\cdot1} + p_{001\cdot0} \\ -1 \end{array}\right\},$$

and

$$\Delta E\{Y(1 \to 0, 0, M_2(0, M_1(1)))\} \leq \min\left\{\begin{array}{l} 3 - p_{000\cdot1} - p_{100\cdot0} - p_{100\cdot1} - p_{001\cdot0} - 2p_{001\cdot1} - 2p_{101\cdot0} - p_{101\cdot1} \\ 1 \\ 2 - p_{100\cdot0} - p_{001\cdot0} - p_{001\cdot1} - p_{101\cdot0} \\ 2 - p_{000\cdot1} - p_{100\cdot0} - p_{100\cdot1} - p_{001\cdot1} - p_{101\cdot0} - p_{101\cdot1} \\ 2 - p_{000\cdot0} - p_{000\cdot1} - p_{100\cdot0} - p_{101\cdot0} \\ 3 - p_{000\cdot0} - 2p_{000\cdot1} - 2p_{100\cdot0} - p_{100\cdot1} - p_{001\cdot1} - p_{101\cdot0} - p_{101\cdot1} \end{array}\right\}.$$

Result 4c:
The bounds given below are valid and tight for $\Delta E\{Y(1 \to 0, 0, M_2(1, M_1(0)))\} = E\{Y(1, 0, M_2(1, M_1(0)))\}- E\{Y(0, 0, M_2(1, M_1(0)))\}$ under Figure 1b.

$$\Delta E\{Y(1 \to 0, 0, M_2(1, M_1(0)))\} \geq \max\left\{\begin{array}{l} -3 + p_{000\cdot0} + p_{100\cdot0} + p_{100\cdot1} + 2p_{001\cdot0} + p_{001\cdot1} + p_{101\cdot0} + 2p_{101\cdot1} \\ -3 + 2p_{000\cdot0} + p_{000\cdot1} + p_{100\cdot0} + 2p_{100\cdot1} + p_{001\cdot0} + p_{101\cdot0} + p_{101\cdot1} \\ -2 + p_{000\cdot0} + p_{100\cdot0} + p_{100\cdot1} + p_{001\cdot0} + p_{101\cdot0} + p_{101\cdot1} \\ -2 + p_{100\cdot1} + p_{001\cdot0} + p_{001\cdot1} + p_{101\cdot1} \\ -2 + p_{000\cdot0} + p_{000\cdot1} + p_{100\cdot1} + p_{101\cdot1} \\ -1 \end{array}\right\},$$

and

$$\Delta E\{Y(1 \to 0, 0, M_2(1, M_1(0)))\} \leq \min\left\{\begin{array}{l} 3 - p_{000\cdot0} - p_{000\cdot1} - p_{100\cdot0} - p_{001\cdot0} - 2p_{001\cdot1} - 2p_{101\cdot0} - p_{101\cdot1} \\ 1 \\ 2 - p_{000\cdot1} - p_{100\cdot0} - p_{100\cdot1} - p_{001\cdot1} \\ 2 - p_{000\cdot1} - p_{001\cdot1} - p_{101\cdot0} - p_{101\cdot1} \\ 2 - p_{000\cdot0} - p_{000\cdot1} - p_{100\cdot0} - p_{001\cdot0} - p_{001\cdot1} - p_{101\cdot0} \\ 3 - p_{000\cdot0} - 2p_{000\cdot1} - 2p_{100\cdot0} - p_{100\cdot1} - p_{001\cdot0} - p_{001\cdot1} - p_{101\cdot0} \end{array}\right\}.$$

Result 4d:
The bounds given below are valid and tight for $\Delta E\{Y(1 \rightarrow 0, 0, M_2(1, M_1(1)))\} = E\{Y(1, 0, M_2(1, M_1(1)))\}-$ $E\{Y(0, 0, M_2 1, M_1(1)))\}$ under Figure 1b.

$$\Delta E\{Y(1 \rightarrow 0, 0, M_2(1, M_1(1)))\} \geq \max \left\{ \begin{array}{l} -1 - p_{000 \cdot 1} - p_{010 \cdot 1} - p_{110 \cdot 1} + p_{001 \cdot 0} + p_{101 \cdot 1} \\ -2 + p_{000 \cdot 0} + p_{000 \cdot 1} + 2p_{100 \cdot 1} + p_{010 \cdot 1} + p_{110 \cdot 1} + p_{101 \cdot 1} \\ -1 + p_{100 \cdot 1} + p_{101 \cdot 1} \end{array} \right\},$$

and

$$\Delta E\{Y(1 \rightarrow 0, 0, M_2(1, M_1(1)))\} \leq \min \left\{ \begin{array}{l} 1 + p_{100 \cdot 1} + p_{010 \cdot 1} + p_{110 \cdot 1} - p_{001 \cdot 1} - p_{101 \cdot 0} \\ 1 - p_{000 \cdot 1} - p_{001 \cdot 1} \\ 2 - 2p_{000 \cdot 1} - p_{100 \cdot 0} - p_{100 \cdot 1} - p_{010 \cdot 1} - p_{110 \cdot 1} - p_{001 \cdot 1} \end{array} \right\}.$$

Result 4e:
The bounds given below are valid and tight for $\Delta E\{Y(1 \rightarrow 0, 1, M_2(0, M_1(0)))\} = E\{Y(1, 1, M_2(0, M_1(0)))\}-$ $E\{Y(0, 1, M_2(0, M_1(0)))\}$ under Figure 1b.

$$\Delta E\{Y(1 \rightarrow 0, 1, M_2(0, M_1(0)))\} \geq \max \left\{ \begin{array}{l} -p_{000 \cdot 0} - p_{000 \cdot 1} - p_{100 \cdot 0} - p_{100 \cdot 1} - p_{010 \cdot 1} - p_{110 \cdot 0} - p_{110 \cdot 1} - p_{001 \cdot 1} - p_{101 \cdot 1} + p_{011 \cdot 0} - p_{011 \cdot 1} \\ -2 + p_{000 \cdot 0} + p_{100 \cdot 0} + 2p_{010 \cdot 0} + p_{110 \cdot 0} + p_{110 \cdot 1} + p_{011 \cdot 0} \\ -1 + p_{010 \cdot 0} + p_{011 \cdot 0} \end{array} \right\},$$

and

$$\Delta E\{Y(1 \rightarrow 0, 1, M_2(0, M_1(0)))\} \leq \min \left\{ \begin{array}{l} 2p_{000 \cdot 0} + 2p_{100 \cdot 0} + 2p_{010 \cdot 0} + p_{110 \cdot 0} + p_{001 \cdot 0} + p_{101 \cdot 0} + p_{011 \cdot 0} - p_{011 \cdot 1} \\ p_{000 \cdot 0} + p_{100 \cdot 0} + p_{010 \cdot 0} + p_{001 \cdot 0} + p_{101 \cdot 0} + p_{011 \cdot 0} \\ 1 - p_{010 \cdot 1} - p_{110 \cdot 0} + p_{001 \cdot 0} + p_{101 \cdot 0} + p_{011 \cdot 0} \end{array} \right\}.$$

Result 4f:
The bounds given below are valid and tight for $\Delta E\{Y(1 \rightarrow 0, 1, M_2(0, M_1(1)))\} = E\{Y(1, 1, M_2(0, M_1(1)))\}-$ $E\{Y(0, 1, M_2(0, M_1(1)))\}$ under Figure 1b.

$$\Delta E\{Y(1 \rightarrow 0, 1, M_2(0, M_1(1)))\} \geq \max \left\{ \begin{array}{l} -p_{000 \cdot 0} - p_{000 \cdot 1} - p_{100 \cdot 0} - p_{100 \cdot 1} - p_{110 \cdot 0} - p_{001 \cdot 0} - p_{001 \cdot 1} - p_{101 \cdot 0} - p_{101 \cdot 1} + p_{011 \cdot 0} - 1 + p_{111 \cdot 1} \\ -2 - p_{000 \cdot 1} - p_{100 \cdot 1} + 2p_{010 \cdot 0} + p_{110 \cdot 0} + p_{110 \cdot 1} - p_{001 \cdot 1} - p_{101 \cdot 1} + p_{011 \cdot 0} \\ -p_{000 \cdot 0} - p_{000 \cdot 1} - p_{100 \cdot 0} - p_{100 \cdot 1} - p_{010 \cdot 1} - p_{110 \cdot 0} - p_{110 \cdot 1} - p_{001 \cdot 0} - p_{001 \cdot 1} - p_{101 \cdot 0} - p_{101 \cdot 1} - p_{011 \cdot 1} \\ -1 - p_{000 \cdot 1} - p_{100 \cdot 1} + p_{010 \cdot 0} - p_{001 \cdot 1} - p_{101 \cdot 1} + p_{011 \cdot 0} \\ -2 + p_{010 \cdot 0} + p_{110 \cdot 0} + p_{110 \cdot 1} + p_{011 \cdot 0} \\ -1 \end{array} \right\},$$

and

$$\Delta E\{Y(1 \to 0, 1, M_2(0, M_1(1)))\} \leq$$
$$\min \left\{ \begin{array}{l} p_{000\cdot0} + p_{000\cdot1} + p_{100\cdot0} + p_{100\cdot1} + p_{010\cdot0} + p_{001\cdot0} + p_{001\cdot1} + p_{101\cdot0} + p_{101\cdot1} + 1 - p_{111\cdot0} - p_{011\cdot1} \\ 1 \\ 1 + p_{000\cdot0} + p_{100\cdot0} + p_{010\cdot0} + p_{001\cdot0} + p_{101\cdot0} - p_{011\cdot1} \\ p_{000\cdot0} + p_{000\cdot1} + p_{100\cdot0} + p_{100\cdot1} + p_{010\cdot0} + p_{001\cdot0} + p_{001\cdot1} + p_{101\cdot0} + p_{101\cdot1} + p_{011\cdot0} \\ 1 + p_{000\cdot0} + p_{100\cdot0} - p_{010\cdot1} + p_{001\cdot0} + p_{101\cdot0} + p_{011\cdot0} \\ 1 + p_{000\cdot0} + p_{000\cdot1} + p_{100\cdot0} + p_{100\cdot1} - p_{010\cdot1} - p_{110\cdot0} + p_{001\cdot0} + p_{001\cdot1} + p_{101\cdot0} + p_{101\cdot1} + p_{011\cdot0} \end{array} \right\}.$$

Result 4g:
The bounds given below are valid and tight for $\Delta E\{Y(1 \to 0, 1, M_2(1, M_1(0)))\} = E\{Y(1, 1, M_2(1, M_1(0)))\} - E\{Y(0, 1, M_2(1, M_1(0)))\}$ under Figure 1b.

$$\Delta E\{Y(1 \to 0, 1, M_2(1, M_1(0)))\} \geq$$
$$\max \left\{ \begin{array}{l} -p_{000\cdot0} - p_{000\cdot1} - p_{100\cdot0} - p_{100\cdot1} - p_{010\cdot1} - p_{001\cdot0} - p_{001\cdot1} - p_{101\cdot0} - p_{101\cdot1} + p_{011\cdot0} - 1 + p_{111\cdot1} \\ -1 - p_{000\cdot0} - p_{000\cdot1} - p_{100\cdot0} - p_{100\cdot1} + p_{010\cdot0} + p_{110\cdot1} - p_{001\cdot0} - p_{001\cdot1} - p_{101\cdot0} - p_{101\cdot1} - p_{011\cdot1} \\ -p_{000\cdot0} - p_{000\cdot1} - p_{100\cdot0} - p_{100\cdot1} - p_{010\cdot1} - p_{001\cdot0} - p_{001\cdot1} - p_{101\cdot0} - p_{101\cdot1} - p_{011\cdot1} \\ -1 - p_{000\cdot1} - p_{100\cdot1} - p_{010\cdot1} - p_{001\cdot1} - p_{101\cdot1} + p_{011\cdot0} \\ -1 - p_{000\cdot1} - p_{100\cdot1} + p_{010\cdot0} - p_{001\cdot1} - p_{101\cdot1} - p_{011\cdot1} \\ -1 \end{array} \right\},$$

and

$$\Delta E\{Y(1 \to 0, 1, M_2(1, M_1(0)))\} \leq$$
$$\min \left\{ \begin{array}{l} p_{000\cdot0} + p_{000\cdot1} + p_{100\cdot0} + p_{100\cdot1} + p_{110\cdot1} + p_{001\cdot0} + p_{001\cdot1} + p_{101\cdot0} + p_{101\cdot1} + 1 - p_{111\cdot0} - p_{011\cdot1} \\ 1 \\ 2 - p_{010\cdot1} - p_{110\cdot0} - p_{110\cdot1} - p_{011\cdot1} \\ p_{000\cdot0} + p_{000\cdot1} + p_{100\cdot0} + p_{100\cdot1} + p_{010\cdot0} + p_{110\cdot0} + p_{110\cdot1} + p_{001\cdot0} + p_{001\cdot1} + p_{101\cdot0} + p_{101\cdot1} + p_{011\cdot0} \\ 1 + p_{000\cdot0} + p_{100\cdot0} - p_{010\cdot1} + p_{001\cdot0} + p_{101\cdot0} - p_{011\cdot1} \\ 2 + p_{000\cdot0} + p_{100\cdot0} - 2p_{010\cdot1} - p_{110\cdot0} - p_{110\cdot1} + p_{001\cdot0} + p_{101\cdot0} - p_{011\cdot1} \end{array} \right\}.$$

Result 4h:
The bounds given below are valid and tight for $\Delta E\{Y(1 \to 0, 1, M_2(1, M_1(1)))\} = E\{Y(1, 1, M_2 1, M_1(1)))\} - E\{Y(0, 1, M_2(1, M_1(1)))\}$ under Figure 1b.

$$\Delta E\{Y(1 \to 0, 1, M_2(1, M_1(1)))\} \geq$$
$$\max \left\{ \begin{array}{l} -2p_{000\cdot1} - 2p_{100\cdot1} - 2p_{010\cdot1} - p_{110\cdot1} - p_{001\cdot1} - p_{101\cdot1} + p_{011\cdot0} - p_{011\cdot1} \\ -1 + p_{010\cdot0} + p_{110\cdot1} - p_{001\cdot1} - p_{101\cdot1} - p_{011\cdot1} \\ -p_{000\cdot1} - p_{100\cdot1} - p_{010\cdot1} - p_{001\cdot1} - p_{101\cdot1} - p_{011\cdot1} \end{array} \right\},$$

and

$$\Delta E\{Y(1 \to 0, 1, M_2(1, M_1(1)))\} \leq$$

$$\min \left\{ \begin{array}{l} p_{000\cdot 0} + p_{000\cdot 1} + p_{100\cdot 0} + p_{100\cdot 1} + p_{010\cdot 0} + p_{110\cdot 0} + p_{110\cdot 1} + p_{001\cdot 0} + p_{101\cdot 0} + p_{011\cdot 0} - p_{011\cdot 1} \\ 1 - p_{010\cdot 1} - p_{011\cdot 1} \\ 2 - p_{000\cdot 1} - p_{100\cdot 1} - 2p_{010\cdot 1} - p_{110\cdot 0} - p_{110\cdot 1} - p_{011\cdot 1} \end{array} \right\}.$$

4 Additional results from examples

estimand	true	estimate (95% CI)	bounds
$\Delta E(Y(1 \to 0, 0, M_2(0))$	-0.09	-0.08 (-0.12 to -0.04)	[-0.36 to 0.90]
$\Delta E(Y(1 \to 0, 0, M_2(1))$	-0.10	-0.13 (-0.18 to -0.08)	[-0.74 to 0.78]
$\Delta E(Y(1 \to 0, 1, M_2(0))$	-0.10	-0.10 (-0.17 to -0.04)	[-1.00 to 0.69]
$\Delta E(Y(1 \to 0, 1, M_2(1))$	-0.10	-0.15 (-0.25 to -0.07)	[-1.00 to 0.32]
$\Delta E(Y(1 \to 0, M_1(0, 0))$	-0.09	-0.09 (-0.12 to -0.05)	[-0.18 to 0.91]
$\Delta E(Y(1 \to 0, M_1(0, 1))$	-0.10	-0.13 (-0.24 to -0.03)	[-0.93 to 0.99]
$\Delta E(Y(1 \to 0, M_1(1, 0))$	-0.10	-0.11 (-0.15 to -0.07)	[-1.00 to 0.61]
$\Delta E(Y(1 \to 0, M_1(1, 1))$	-0.10	-0.15 (-0.28 to -0.04)	[-1.00 to 0.40]
$\rho E(Y(1 \to 0, 0, M_2(0))$	0.05	0.08 (0.00 to 0.40)	-
$\rho E(Y(1 \to 0, 0, M_2(1))$	0.03	0.08 (0.00 to 0.32)	-
$\rho E(Y(1 \to 0, 1, M_2(0))$	0.04	0.05 (0.00 to 0.16)	-
$\rho E(Y(1 \to 0, 1, M_2(1))$	0.03	0.04 (0.00 to 0.18)	-
$\rho E(Y(1 \to 0, M_1(0, 0))$	0.05	0.12 (0.00 to 0.50)	-
$\rho E(Y(1 \to 0, M_1(0, 1))$	0.03	0.02 (0.00 to 0.07)	-
$\rho E(Y(1 \to 0, M_1(1, 0))$	0.04	0.11 (0.00 to 0.35)	-
$\rho E(Y(1 \to 0, M_1(1, 1))$	0.03	0.02 (0.00 to 0.08)	-

Table 2: Numeric values for the COVID example. The rows starting with ρ present the ratios of probabilities rather than the differences.

References

Rhian M Daniel, Bianca L De Stavola, SN Cousens, and Stijn Vansteelandt. Causal mediation analysis with multiple mediators. *Biometrics*, 71(1):1–14, 2015.
